# Intra- and intersexual differences in parasite resistance and female fitness tolerance in a polymorphic insect

**DOI:** 10.1098/rspb.2016.2407

**Published:** 2017-01-25

**Authors:** Beatriz Willink, Erik I. Svensson

**Affiliations:** Department of Biology, Evolutionary Ecology Unit, Lund University, Ecology Building, Lund 223-62, Sweden

**Keywords:** frequency-dependence, parasites, polymorphism, resistance, sexual conflict, tolerance

## Abstract

To understand host–parasite interactions, it is necessary to quantify variation and covariation in defence traits. We quantified parasite resistance and fitness tolerance of a polymorphic damselfly (*Ischnura elegans*), an insect with three discrete female colour morphs but with monomorphic males. We quantified sex and morph differences in parasite resistance (prevalence and intensity of water mite infections) and morph-specific fitness tolerance in the females in natural populations for over a decade. There was no evidence for higher parasite susceptibility in males as a cost of sexual selection, whereas differences in defence mechanisms between female morphs are consistent with correlational selection operating on combinations of parasite resistance and tolerance. We suggest that tolerance differences between female morphs interact with frequency-dependent sexual conflict, which maintains the polymorphism locally. Host–parasite interactions can therefore shape intra- and intersexual phenotypic divergence and interfere with sexual selection and sexual conflict.

## 1. Background

Phenotypic traits may become negatively associated when they influence the strength of selection on each other by being costly and functionally redundant [1,2]. This emergent form of phenotypic covariation—i.e. the expression of one advantageous trait or another but not both—is often a result of correlational selection, which favours suites of co-adapted traits resulting in phenotypic and/or genetic correlations between them [3–5]. For example, negative trait correlations between resistance and tolerance, two conceptually different defence mechanisms, have long been acknowledged by plant scientists [6–17]. Natural selection for resistance (i.e. adaptations that reduce herbivore or pathogen burden) results in lower damage, which in turn weakens selection for tolerance (i.e. the ability to mitigate the fitness consequences of natural enemies or antagonists) [13]. Conversely, the evolution of tolerance reduces the fitness costs of antagonistic interactions, thereby decreasing the benefits of resistance [8,11,12,14].

More recently, the idea that functional redundancy and correlational selection may limit the simultaneous expression of defence traits has also been applied to research on enemy–victim interactions in animals [18], including host defences against parasites [19], brood parasites [20] and alternative mating tactics in females that defend themselves against male-mating harassment in the context of sexual conflict [21]. However, empirical evidence for negative associations between resistance and tolerance remains limited in animals [18,22], partly owing to the difficulties of elucidating the mechanisms of and genetic variation in the tolerance component of defences. Quantifying variation in tolerance is empirically challenging because the reaction norm of an organism in relation to the intensity of the antagonistic interaction needs to be characterized [23]. In the case of parasitism, multiple genotypes or lines should be naturally or experimentally exposed to parasites to quantify such reaction norms [7,19,23]. Consequently, the vast majority of previous research on tolerance variation and the relationship between resistance and tolerance has been conducted in laboratory settings and on a few model organisms [19,24–27], whereas only a few empirical studies have attempted to assess tolerance variation in natural animal populations [20,28].

Immunity often differs among discrete phenotypic categories, including the two sexes [29–33] (i.e. sexual dimorphism), heritable trophic polymorphisms or ‘ecotypes’ [34] and genetically determined colour morphs [35–38]. The existence of such trait differences suggests phenotype-dependent trade-offs between reproduction and defence against parasites and pathogens [30,31]. For instance, compared with females, males might sacrifice health and longevity for mating, leading to sex differences in immunity and/or parasite avoidance [30]. However, even if such different optima exist for the sexes or other discrete phenotypic categories, intralocus conflict [39] will limit phenotypic divergence in all sexually reproducing organisms [29,32,37]. Intralocus conflict is thus ubiquitous and not restricted to constraining sexual dimorphism [39,40]. It can also limit trait divergence between other discrete sympatric phenotypic categories, including phenotypic divergence between heritable colour morphs [35,37,41].

Here, we compare defences against parasitic water mites (Acari:Arrenuridae) by males and females belonging to three different heritable colour morphs of a pond damselfly (*Ischnura elegans*). Sexual conflict over mating rates plays a key role in the maintenance of such female-limited colour polymorphisms, which are common in many species of temperate pond damselflies of the family Coenagrionidae [42–45]. Among the female morphs, there is typically one type of females which are male-coloured (‘androchrome’ females, or andromorphs), and which are thought to be male mimics that avoid costly male-mating harassment through visual deception [21,46]. The different female morphs differ in a number of phenotypic traits apart from coloration, including aggressiveness, fecundity and developmental time [47–50]. Because morphs have diverged in multiple traits and these differences might often be adaptive, polymorphic damselflies are well suited for investigating if inter-morph genetic correlations constrain morph divergence [41], especially in immunity traits, which are often linked to coloration in insects [51].

We analysed a large field dataset on parasite loads on males and females that have been collected for over a decade. These field data come from more than a dozen natural populations of *I. elegans* in southern Sweden. First, we compared parasite loads of males and the three female colour morphs. Based on sexual selection theory, we expected males to have higher parasite loads than females [30,31]. We further expected the androchrome females, that are ‘masculinized’ in body colour and are similar to males in morphology, physiology and behaviour [49], to have high parasite loads that should be comparable to males, thus higher than the two other female morphs. Second, we investigated if resistance and tolerance differed between female morphs, as we would expect if correlational selection couples the defence components owing to the existence of different adaptive peaks. To answer this question, we quantified morph and population differences in resistance and tolerance and investigated how alternative combinations of defence mechanisms influence female fitness consequences of parasitic mites. Correlational selection predicts a negative association between resistance and tolerance between morphs. As we expected androchrome females to be more heavily parasitized owing to their morphological and physiological resemblance to males (see above), we also predicted that this morph should be relatively tolerant in comparison to more resistant females.

## 2. Material and methods

### (a) Study system

The common bluetail (*I. elegans*) is an abundant damselfly distributed throughout the Palaearctic [52]. The northern end of the species distribution extends to the southern third of Sweden, where *I. elegans* is univoltine. Adults emerge between late May and early August and the aquatic nymphs overwinter. Adult males are monomorphic in colour while females occur in three heritable and discrete colour morphs [53]. Female colour morph development is governed by three alleles in a dominance hierarchy at a single polymorphic autosomal locus [54]. There are thus six genotypes, but only three visually discernible phenotypes [54]. Androchrome females carry at least one copy of the most dominant allele and are male-coloured, displaying the same blue markings and melanin patterning as males. *Infuscans* females, which can develop from two different genotypes, have yellow-greenish thorax markings instead of blue and deposit melanin on the eighth abdominal segment, where androchrome females exhibit a blue patch similar to males [53]. Finally, *Infuscans-obsoleta* females are homozygous for the most recessive allele, have weak melanin patterning and are reddish-brownish in coloration. For simplicity, we will hereafter denote these three female morphs as A-, I- and O-females, respectively.

Water mites of the genus *Arrenurus* are among the most common external parasites of odonates [55,56]. They attach to damselfly nymphs but remain phoretic until the time of emergence, when they can perforate the cuticle and start feeding on their host's fluids [57]. Water mite prevalence may vary markedly throughout the breeding season [58], but they can be more prevalent and numerous in females than males in some species [56]. In another species of pond damselfly (*Coenagrion puella*), water mites increase condition-dependent mortality in both sexes and reduce female fecundity [59].

### (b) Data collection

We used data from our long-term population study on *I. elegans* [21,43,45]. This long-term study encompasses regular surveys of more than a dozen damselfly populations and up to 17 generations, as *I. elegans* has one discrete generation per year in southern Sweden. We started collecting parasite data during the breeding season of 2003, and we have continued with this until 2016. Fecundity data are available for the period between 2003 and 2016, except for 2010 and 2012, when we only have parasite data. A total of 16 populations, distributed over an area of 40 × 40 km were included in this study. Owing to local extinctions, two populations were not sampled in the last 2–3 years and one and three new populations, respectively, were added in 2011 and 2012. Every year, populations were visited on several occasions (mean number of visits ±s.d. = 5.8 ± 2.6, range = 1–15) during the summer months. Field work usually started in early June, depending on local weather conditions, and continued into the first few days of August.

Damselflies were caught using hand nets and transported into an indoor laboratory, where they were classified with respect to sex, colour morph (in females) and the number of water mite parasites on each sexually mature individual. We also noted whether the damselflies were in copula at the moment of collection. Females which were caught in copula were set in individual cups for oviposition (except in 2010 and 2012), and were provided with moistened filter paper where eggs could be attached. After 72 h, females were released and the eggs were counted. This gave us a cross-sectional and instantaneous fecundity estimate, a fitness component that is correlated with lifetime female fecundity [43,57]. Note that as females were not provided with food during the oviposition procedure, all eggs that were laid should reflect feeding, general weather conditions and mating interactions that these females experienced the preceding days in their local field populations.

### (c) Statistical analysis

We analysed resistance and tolerance data separately, with Bayesian generalized mixed models (GLMM) with Markov chain Monte Carlo (MCMC) estimation, using package ‘MCMCglmm’ [60] in R [61]. Our response variables were: (i) water mite counts in all sampled individuals for the analysis of resistance, and (ii) fecundity of mated females as a fitness component for the analysis of tolerance. Because in both cases the response variables were highly over-dispersed counts, we specified the Poisson family in package MCMCglmm, which uses an additive model for over-dispersion.

To analyse differences in parasite resistance, we used a zero-altered Poisson (ZAP) GLMM. ZAP models contain two sub-models: one related to the zeroes and one related to counts greater than zero. Therefore, each outcome (i.e. datum) depends on two latent variables: (i) the probability (in logit scale) of the response variable being non-zero, which is here interpreted as the probability of having any parasites at all, and (ii) the mean parameter of a zero-truncated Poisson distribution [62], which in this case corresponds to the estimated parasite count in parasitized individuals. Thus, for each female morph as well as for the males we obtained an estimate of the probability of parasitism (hereafter prevalence) and the mean number of parasites in parasitized individuals (hereafter intensity). We chose this model because most damselflies in our study (nearly 84%) had no parasites. Moreover, using ZAP distribution models is useful when two different processes underlie the probability and the expectation of an outcome. For instance, in this case prevalence might be mainly determined by exposure to mites in the aquatic stage, whereas infection intensity at a given prevalence is instead more likely to reflect immunity-mediated resistance. We included a population-by-year interaction as a random effect on both the logit and Poisson processes to account for spatio-temporal variation in these processes.

For females, we had access to fecundity data that we used to quantify tolerance. Tolerance is inversely proportional to the reduction in fitness with increasing infection intensity. To estimate morph-specific tolerance, we evaluated the effects of parasites on female fecundity, using a GLMM with Poisson error distribution and fitted by MCMC as described above. In this analysis, a difference in slopes between the heritable morphs would indicate a morph effect on tolerance. Populations and cohorts may also differ in their intercepts (fecundity in the absence of parasites) and in their sensitivity to water mite infections. We accounted for statistical non-independence owing to population of origin and season by fitting both random intercepts and slopes for each combination of population and season. To do this, we specified the unstructured variance function in MCMCglmm, which estimates a 2 × 2 matrix including the random-effect variance in intercepts and slopes and the covariance between them. We only estimated tolerance for A- and I-females, which together represented more than 95% (*N*_A_ = 2517, *N*_I_ = 1303) of all females found in copula. The O-females (*N*_O_ = 178) were too rare to reliably account for spatio-temporal variance in reaction norms.

For both models, we used uninformative priors with a low degree of belief in all parameters (see the electronic supplementary material, Supporting Methods). The models were run for 2 000 000 iterations preceded by a burn-in of 100 000 iterations and saving every 1000th iterations to avoid autocorrelation between draws (autocorrelations were weaker than 0.10 for all variance components). This resulted in an effective sample size of 2000 iterations. We evaluated model convergence visually by plotting the chains and checking that they had mixed properly and by plotting the autocorrelation between draws (electronic supplementary material, figures S1–S5). Also, we used the Gelman–Rubin convergence diagnostic in the ‘coda’ R package [63] (electronic supplementary material, Supporting Methods). We report here the mean of the posterior density distribution of model parameters with 95% credible intervals (CI), which indicate the precision of an estimate. *P*-values for comparisons between levels are given by the proportion of iterations where one level has a larger or smaller estimate than the other. *p*-values for correlations are given by the proportion of iterations where the regression coefficient between two variables is above or below zero.

As these analyses revealed morph differences in both resistance and tolerance (see Results), we proceeded by investigating how these defences jointly influenced the fitness consequences of water mite parasitism. Virulence is here defined as the parasite-induced reduction in host fitness considering both defence mechanisms [23]. Here, we have modelled the effects of water mites as exponentially decreasing female fecundity with increasing infection intensity. Therefore, virulence was calculated as the proportional reduction in fecundity in response to morph-specific parasite loads:2.1
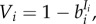
where *V_i_* is virulence experienced by morph *i*, *I_i_* is the morph effect on infection intensity and *b_i_* is the base parameter of the exponential function describing morph tolerance. Because *I* and *b* were estimated with error in previous models, we re-sampled 2000 iterations of the marginal posteriors of these coefficients to calculate virulence. As described above, we report as *p*-values the proportion of iterations in which one morph has a higher (or lower) virulence than the other.

## 3. Results

**Figure 1.**
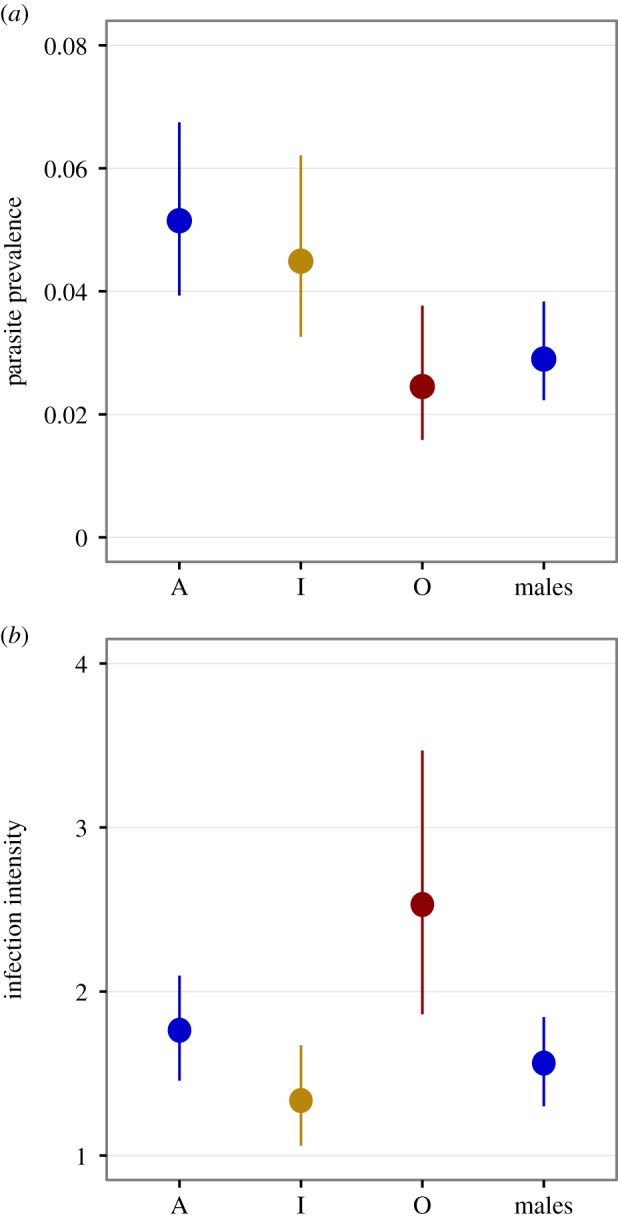
Resistance to parasitic water mites by males and the three heritable female morphs of *I. elegans*. (*a*) Prevalence as the probability of harbouring at least one water mite. (*b*) Infection intensity as the number of mites in each parasitized individual. Symbols represent MCMCglmm posterior means and 95% credible intervals. A, androchrome females; I, *Infuscans* females; O, *Infuscans-obsoleta* females. (Online verison in colour.)

We obtained data on parasite loads from 26 677 adult damselflies: 5517 A-females, 1856 I-females, 518 O-females and 18 786 males. Both the prevalence and intensity of water mite infections varied significantly among the four phenotypes (figure 1). A- and I-females had similar parasite prevalence (pMCMC = 0.219) and both of these female morphs were more likely to be parasitized by mites than O-females (A versus O pMCMC < 0.001, I versus O pMCMC = 0.004). However, among parasitized individuals, A-females harboured greater parasite numbers than I-females (pMCMC = 0.003) and O-females had even greater infection intensities than A-females (pMCMC = 0.009). Unexpectedly, males had lower parasite prevalence than both A- and I-females (both pMCMC < 0.001), and intermediate infection intensity (A versus males pMCMC=0.019, I versus males pMCMC = 0.057; figure 1).

**Figure 2.**
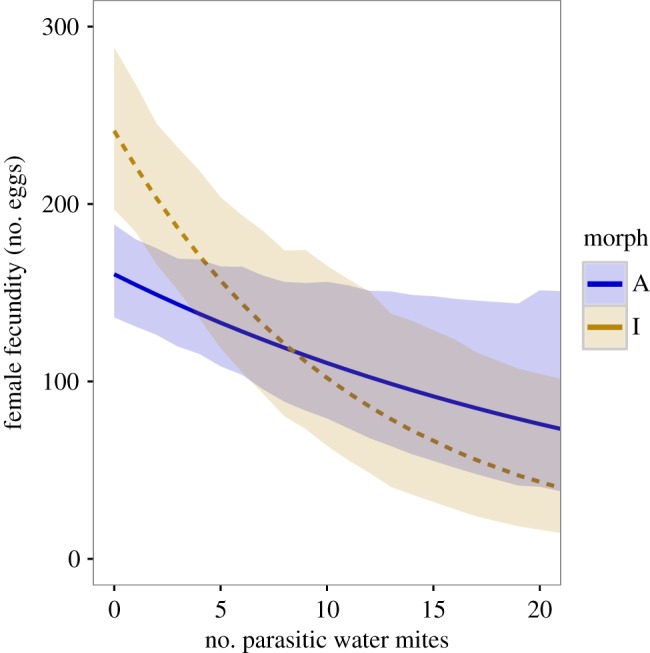
Fecundity tolerance to parasitic mites among heritable colour morphs in females of *I. elegans*. Tolerance is here defined as a slope and a measure of the reaction norm of female fecundity to parasite load. Parasite load ranged from 0 to 56. We plot the tolerance response over more than 98% of the range of infection intensities in natural populations of *I. elegans* in southern Sweden. The fitted lines represent the predictions of the effects of water mites on female fecundity and the shaded areas cover the 95% credible intervals.

**Figure 3.**
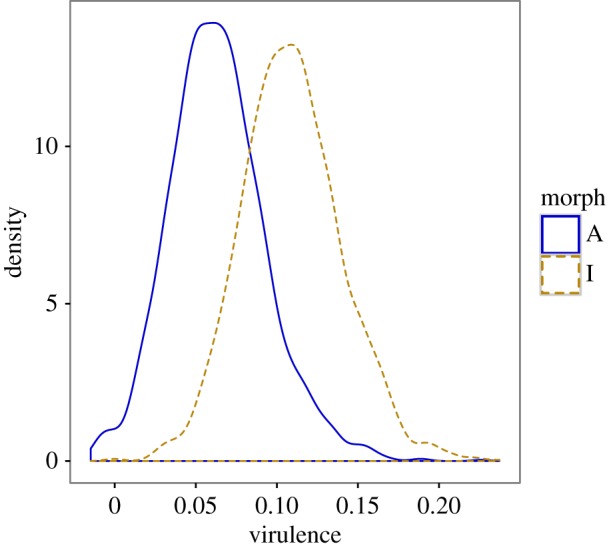
Morph-specific virulence in females of *I. elegans* as the proportional reduction in fecundity owing to water mite infections. Kernel density plots show the overlap between the distributions of 2000 estimates for each morph-specific coefficient. (Online version in colour.)

Fecundity tolerance differed significantly between the two most common morphs (figure 2; electronic supplementary material, S6 and S7). A-females were less sensitive than I-females in their fecundity response to water mite infections (pMCMC = 0.029, figure 2). In A-females, each additional water mite caused a mean decrease in egg production of 3.7% (95% CI = 0.5–6.9%), whereas in I-females the proportional decrease was more than two times higher (mean = 8.3%, 95% CI = 4.0–12.6%). The intercepts of these regressions also differed significantly (mean difference = 80.0, 95% CI = 36.3–129.0 pMCMC < 0.001). In the absence of parasites, I-females had 33% higher estimated fecundity than A-females. Despite differences in defence mechanisms, both morphs had similar effects on parasite virulence (pMCMC = 0.288; figure 3).

There was pronounced and significant spatio-temporal variation in the prevalence and intensity of water mite infections (electronic supplementary material, table S2 and figure S8). The random effects of populations and years accounted for nearly two-thirds of the variance in prevalence (mean = 65.6%, 95% CI = 60.2%–71.7%) and a third of the variance in infection intensity (mean = 36.6%, 95% CI = 29.4%–43.6%). In striking contrast with this, between-population variance in the slope of the fecundity response to water mites was negligible (mean = 0.003, upper 95% CI = 0.008), and the random variance in the intercept accounted for a modest 5.2–11.3% of all variation in female fecundity (electronic supplementary material, table S3 and figure S9).

## 4. Discussion

Results in this study allowed us to compare infection prevalence and intensity between discrete phenotypes: the three female colour morphs and the monomorphic males (figure 1). The traditional view among sexual selection researchers that have studied vertebrates is that males should generally suffer greater parasitism than females, either because of sex-specific parasite exposure or because of sex-differences in physiological immunosuppression [30,64]. This view has been empirically challenged by findings of high female parasitemia in arthropods [65], including damselflies [56], and in some vertebrates [66]. Here, we found that males did not have the highest parasite prevalence or intensity (figure 1) and that resistance differences within one sex (i.e. between female morphs) were as pronounced as differences between males and females (figure 1). Thus, ecological and physiological differentiation and alternative defence strategies can occur both between the sexes [31,66–68], but also between other heritable phenotypic categories [36], including female colour morphs (this study).

Sex and morph differences in parasite prevalence and intensity show that intra-populational divergence between the sexes as well as between different morphs within females has partly overcome the genetic constraints set by intralocus conflict, which should constrain phenotypic divergence and sexual dimorphism [39]. Previous sex-difference generalizations might have been overly simplistic, as intrasexual differentiation can also be pronounced, particularly in polymorphic systems with alternative mating and reproductive strategies (cf. [46]). Presumably, these phenotypic differences in parasite prevalence and infection intensity reflect differences in life-history trade-offs between the sexes and between morphs within females. Higher infection intensity for two of the three female morphs in this study is notable in the light of empirical evidence for higher investment in some components of immunity in female insects, compared with males [31]. The discordance between these results and previous theory calls for both new theory and more empirical research on how life-history trade-offs, sexual selection, genetic architecture and host–parasite coevolution can jointly shape sexual dimorphism in defence traits [29,30,32,33,37,40,69].

We have also found that the two most common heritable female morphs in *I. elegans* use different combinations of defence strategies against parasitic mites (figures 1–2)*.* More specifically, A-females have high parasite loads but are relatively tolerant to these parasites in terms of reductions in their fecundity, while the more resistant I-females pay higher fecundity costs of harbouring many parasitic mites. We were not able to estimate the tolerance response in O-females with precision, as this morph represented only about 5% of all mating females in our study populations. O-females seem to be poorly defended, having the highest infection intensities when they become parasitized (figure 1). As this O-morph is rare in southern Sweden [70] and our estimates are consequently uncertain, we focus below on the differences between the much more common A- and I-females.

The morph differences in host resistance and tolerance are consistent with heritable colour morphs frequently being subject to multifarious variation in physiological, life-history and behavioural traits [35–39,71,72]. In *I. elegans,* the correlated expression of colour patterning and defence strategies can also been seen in the context of sexual conflict over male-mating harassment of females [21]. It takes more clasping attempts for a male to coerce an I-female into copulation, but this higher resistance in I-females is in turn associated with a greater reduction in fecundity compared with A-females [21,73]. The results in this study, in combination with our previous research in this system, show that I-females have similar and high sensitivity in terms of their fitness towards two different antagonistic biotic agents: males and parasites. In other insects, heritable colour morphs often differ in several fitness-related traits, for instance habitat preferences [74] and anti-predator behaviours [75], but the mechanistic basis of such trait correlations is not fully understood [76].

One proximate mechanism causing this negative association between resistance and tolerance in *I. elegans* could involve differential regulation of melanin, as melanin has pleiotropic effects on immunity, development, mating behaviour and female fecundity [77,78]. Water mite resistance by melanotic encapsulation depends on the activity of phenoloxidases (POs), which are a major component of insect humoral immunity [79]. PO expression mediates the link between immunity and discrete differences in melanization in other insects [80]. This mechanism could at least partially account for the colour distinctiveness between *I. elegans* morphs, given that A- and I-females differ in the extent of cuticle melanization formed during post-emergence development. Moreover, juvenile hormones, which stimulate oocyte maturation and vitelogenesis [81,82], downregulates the expression of POs [83]. Across insects (including other damselflies) there is empirical evidence for fitness costs of immunity caused by an antagonistic relationship between melanotic encapsulation and reproduction [83–87]. Thus, regulation of PO activity might potentially explain variation in both tolerance and resistance. Future research on the molecular basis and genetic architecture of this and other colour polymorphisms are needed to clarify the mechanistic link between differences in coloration and parasite defences.

Hormonal regulation, as a mechanism by which a limited number of loci control the expression of co-selected traits, is one possible outcome of correlational selection [88]. However, correlational selection alone is not sufficient to maintain sympatric genetic variation in defence components. Our results show that the net effect of the different combinations of defence mechanisms between these morphs results in similarly experienced levels of virulence (figure 3), but they do not provide any direct evidence for the suggestion that water mites *per se* contribute to the maintenance of this genetic polymorphism within local populations [56]. If water mites were the only selective pressure acting on these morphs, local parasite-mediated selection should result in the fixation of I-females in populations with few parasites, owing to a cost of tolerance in benign environments, as shown by the intercept difference between morphs (figure 2). Conversely, A-females would benefit in populations with many parasites, as the reaction norms of these morphs cross at high parasite pressures, which would be expected to result in the fixation of these A-females (figure 2). We suggest that the maintenance of morph differences in host defences against water mites is most likely a by-product of negative frequency-dependent selection (NFDS) on female morphs through intersexual interactions [43,45].

There is substantial evidence for NFDS on female fecundity in female-polymorphic species of damselflies. Males develop a search image for common morphs, which results in higher male pre-mating harassment and reduced female fecundity [21,42,89]. In *I. elegans*, the power of NFDS in maintaining this polymorphism is illustrated by the fact that across 90 European populations of this species, none is monomorphic [70], and across 16 populations in southern Sweden, morph frequencies fluctuate significantly less between generations than expected from genetic drift alone [45]. While NFDS influences morph-frequency dynamics within local populations, between-population differences in morph frequencies are mainly influenced by other ecological factors, including temperature and microclimatic variation [70,90] and possibly also parasite pressure (this study). This tension between the conservative role of NFDS that maintains morphs locally and parasites that could increase population divergence in morph frequencies might result in a geographical coevolutionary selection mosaic between parasites and female morphs at the landscape scale (cf. [91]) that merits future investigation.

Morph differences in parasite tolerance are also interesting because of the positive selective effect that tolerance is expected to have on its own frequency. While variation in resistance can persist simply owing to its negative effect on parasite prevalence, which in turn selects for reduced resistance [92], the positive ecological feedback caused by tolerance should drive its fixation [93,94] unless tolerance comes at a fitness cost, say in host lifespan or fecundity [95]. Tolerance can also relax selection on hosts to oppose transmission, increasing pathogen prevalence [96], and potentially leading to more disease-induced mortality [97]. Here, we found that between-population variance in tolerance was negligible, in line with previous studies on host genotypes or families of various arthropods [94,98]. Within local populations of *I. elegans*, the negative pullback force of NFDS in sexual conflict may indirectly influence host–parasite dynamics and limit parasite prevalence by reducing the frequency of the more susceptible but tolerant A-females.

## 5. Conclusion

Resistance and tolerance to parasitic mites are inversely associated in the two most common female morphs of *I. elegans*, consistent with correlational selection favouring different combinations of defence components in these different morphs. Compared with host resistance, tolerance has received considerably less attention in the animal literature [18,99]. To our knowledge, the data presented here are the first evidence of genetically associated tolerance variation in natural populations of an invertebrate host. With increasing infection intensity, variation in tolerance between these morphs will decrease their intrinsic fecundity differences (cf. intercept in figure 2). Unlike resistance, morph-specific tolerance is stable across populations and persists within populations owing to the role of NFDS that maintains this female colour polymorphism. NFDS might therefore indirectly influence tolerance variation as a correlated response to selection on the female morphs. We emphasize the importance of quantifying variation in both resistance and tolerance in natural populations to better understand how defence traits influence host fitness, host–parasite coevolutionary dynamics and the genetic composition of host populations.

## Supplementary Material

Supplementary Methods and Results

## Data accessibility

Data available from the Dryad Digital Repository: http://dx.doi.org/10.5061/dryad.65451 [100].
