# Supplementary material for: Intra- and intersexual differences in parasite resistance and female fitness tolerance in a polymorphic insect
Source: Proc Biol Sci. 2017 Jan 25;284(1847):20162407. doi: 10.1098/rspb.2016.2407 (PMC5310041; doi:10.1098/rspb.2016.2407)
Supplement: Supplementary Methods and Results [file rspb20162407supp1.pdf]

Electronic Supplementary Information (ESM)

Intra- and intersexual differences in parasite resistance and female fitness tolerance in a polymorphic insect

Beatriz Willink*, Erik I Svensson

Evolutionary Ecology Unit, Department of Biology, Lund University, Lund, Sweden

*Corresponding author email: beatriz.willink@biol.lu.se

Supplementary Methods

1. Morph variation in resistance

Priors: In zero-altered MCMCglmm the residual variance of the zero-deflation is fixed at 1 [1], and in this case the covariance between fixed effect levels was also set to zero. Thus the prior for the residual variances was specified as a diagonal matrix for the logit and Poisson processes (V = diag(2)) with fixed variances (fix = 2) and low degree of belief in all parameters (nu = 0.002). Priors for random effects are based on an inverse Wishart distribution [1], in this case with V = 1 and nu = 0.002 for both variance components.

Diagnostics: We performed visual diagnostics of the mixing properties and convergence of three independent chains (Fig. S1). The Gelman-Rubin diagnostic of these runs also indicates convergence (multivariate PSRF = 1.10). Autocorrelation between saved iterations was low for all variance components (Fig S2). The final autocorrelations were 0.041 for the logit process and 0.069 for the Poisson process, and 0.008 for the residual variation in the count process.

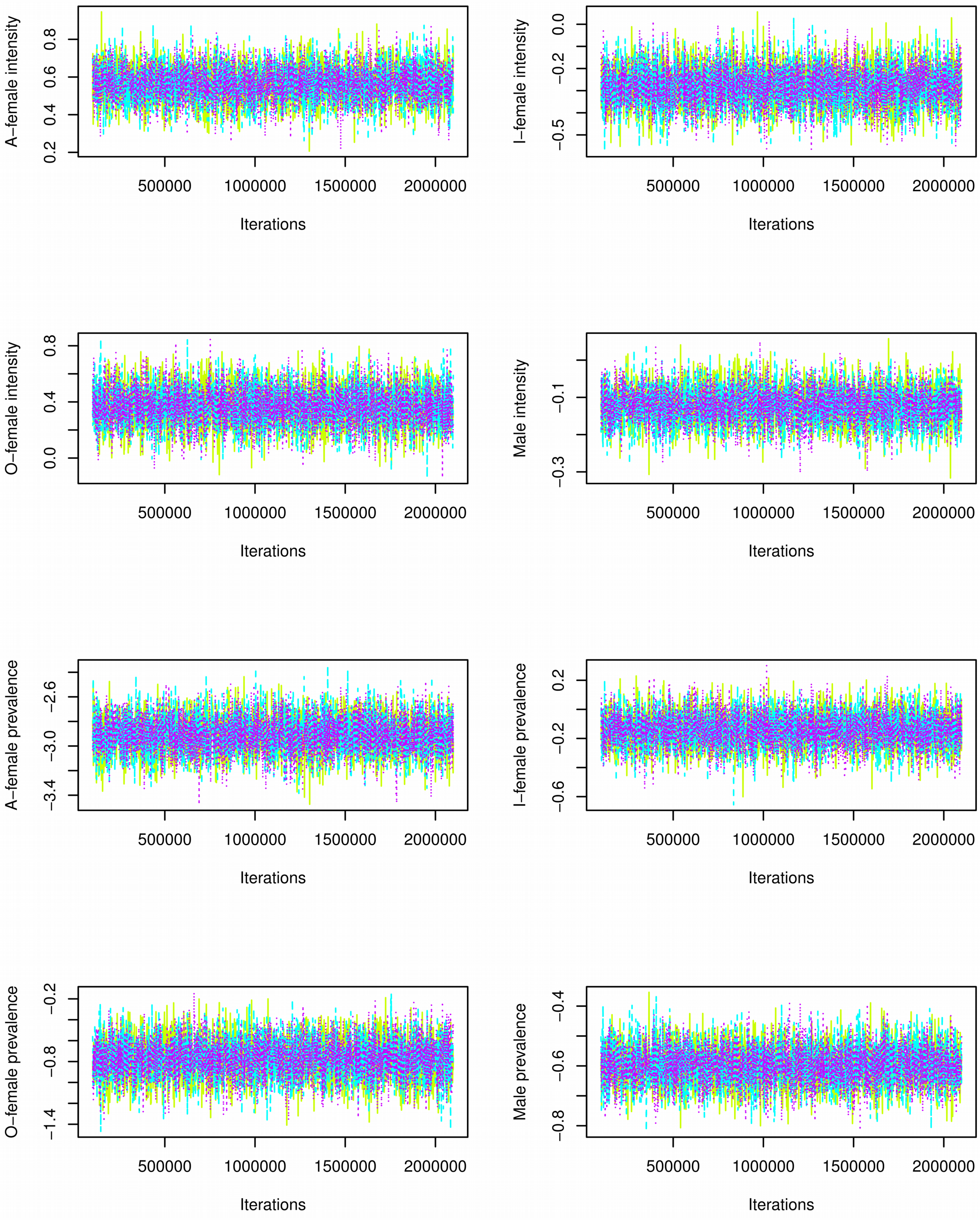
A−female intensity
0.2
0.4
0.6
0.8
I−female intensity
−0.5
−0.2
0.0
O−female intensity
0.0
0.4
0.8
Male intensity
−0.3
−0.1
A−female prevalence
−3.4
−3.0
−2.6
I−female prevalence
−0.6
−0.2
0.2
O−female prevalence
−1.4
−0.8
−0.2
Male prevalence
−0.8
−0.6
−0.4
500000
1000000
1500000
2000000
Iterations

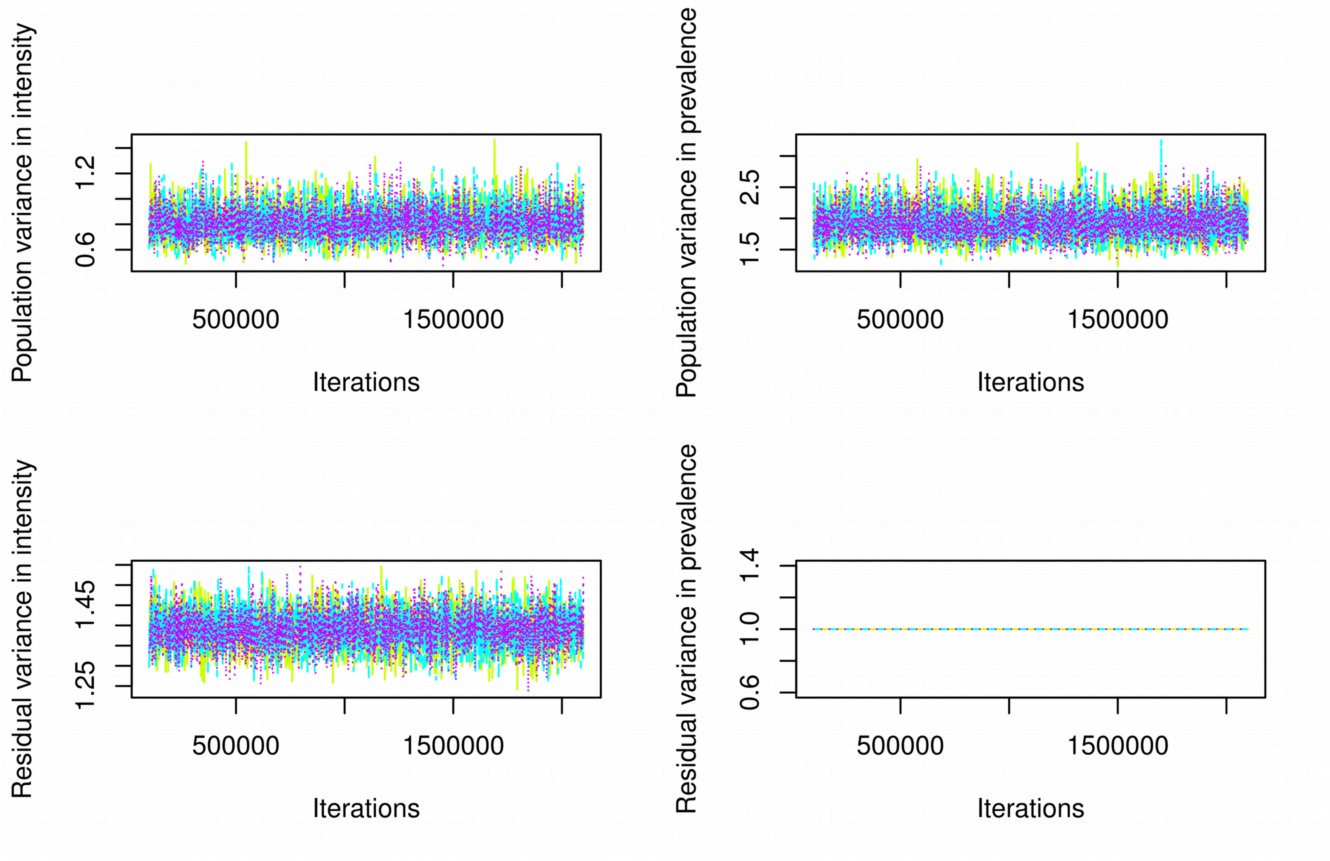

Fig. S1. MCMC summary plots of three Markov chains for each fixed-effect coefficient and variance component of the zero-altered glmm. The fixed-effect coefficients correspond to the mean parameter of a zero-truncated Poisson distribution for each damselfly morph (infection intensity), and the zero-deflation estimate for each morph (parasite prevalence). The random-effect coefficients represent the spatial and temporal variance in these parameters. The response variable is the number of parasitic mites on host damselflies.

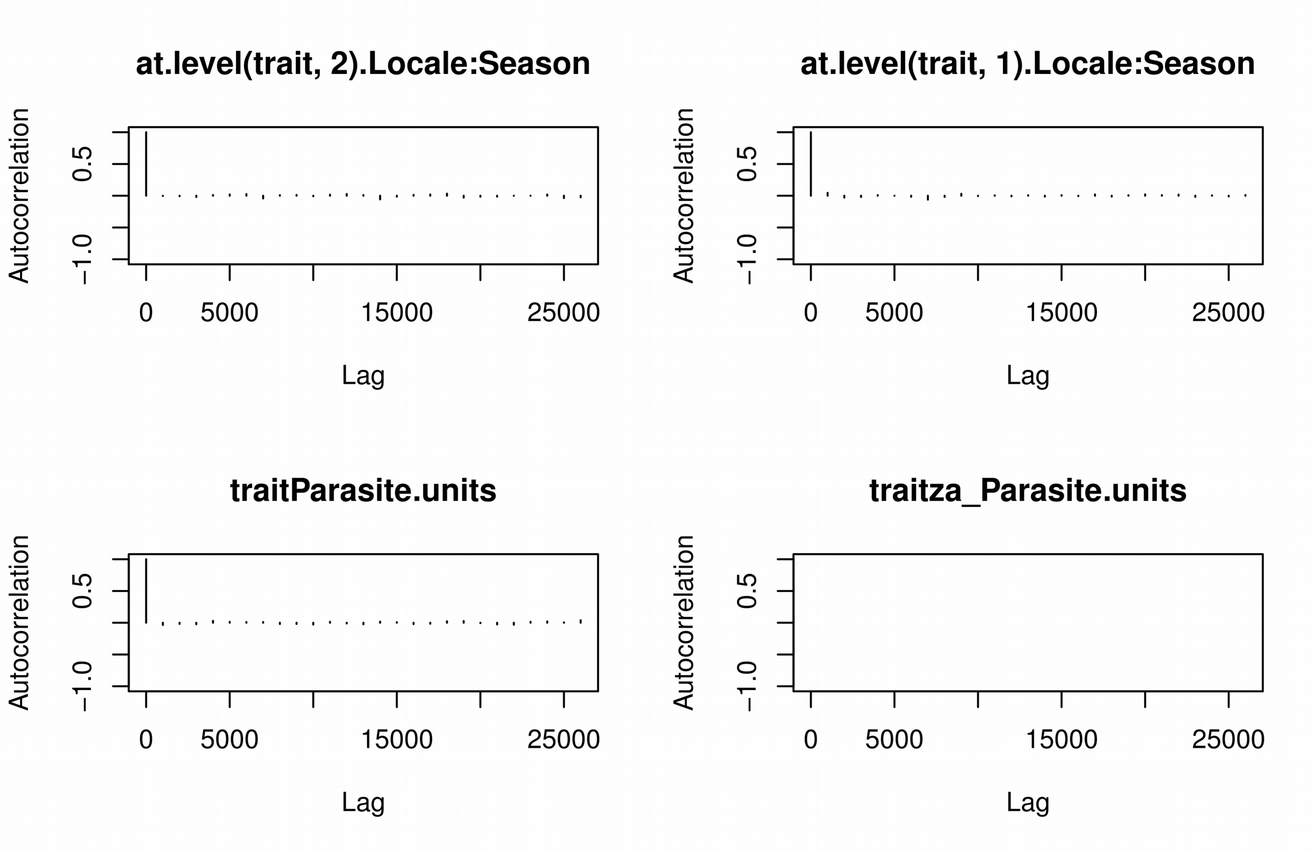

Fig. S2. Autocorrelation plots for the zero-altered MCMCglmm estimates of inter-population variance in parasite prevalence (trait, 2) and infection intensity (trait,1) and residual variance (units) in infection intensity. Residual variance in the binomial process is fixed to 1.

2. Morph variation in tolerance

Priors: We started with the specification of inverse Wishart priors on the fixed effects, variances and covariances, with variance at the limit set to 1 and low degree of belief (nu=0.002). We repeated the analysis using, improper priors which, unlike the inverse Wishart are non-informative for the residual variance (V = 0 and nu = -2). Improper priors can produce a run into numerical and inferential problems (i.e. a reducible chain) but have other useful properties and can be applied when there is enough replication for the posterior to be defined [1]. We obtained the same results with these two prior specifications (Fig S6), indicating that the results in our first analysis were not driven by the priors.

We also used less informative priors for the (co) variances, some of which appeared to be close to zero in our first analysis. Parameter-expanded priors can be less informative as well as improve mixing and speed up convergence in the chains of variances close to zero. These methods use additional priors for redundant parameters that are not identified by the likelihood, and thereby can influence the distribution of variance components. The priors on these parameters come from a non-central scaled F distribution and call for a mean and scale [2]. We used a proper Cauchy prior for the standard deviation, setting the the mean to 0 and alpha.V = diag(2)*1000. Using these priors did not change any of the fixed effect estimates, but resulted in a right skewed posterior distribution for the inter-population variance in slope (Fig. S6), further decreasing support for inter-population variance in tolerance.

Diagnostics: Visual diagnostics of three independent runs of indicate good mixing and chain convergence for all fixed-effect coefficients and variance components (Fig. S7). The Gelman-Rubin also shows convergence (multivariate PSRF = 1.08). The autocorrelations between draws for spatial-temporal (co)variance estimates were for the 0.018 intercept, 0.022 for the slope of the regression curve and 0.003 for the covariance between them (Fig. S8).

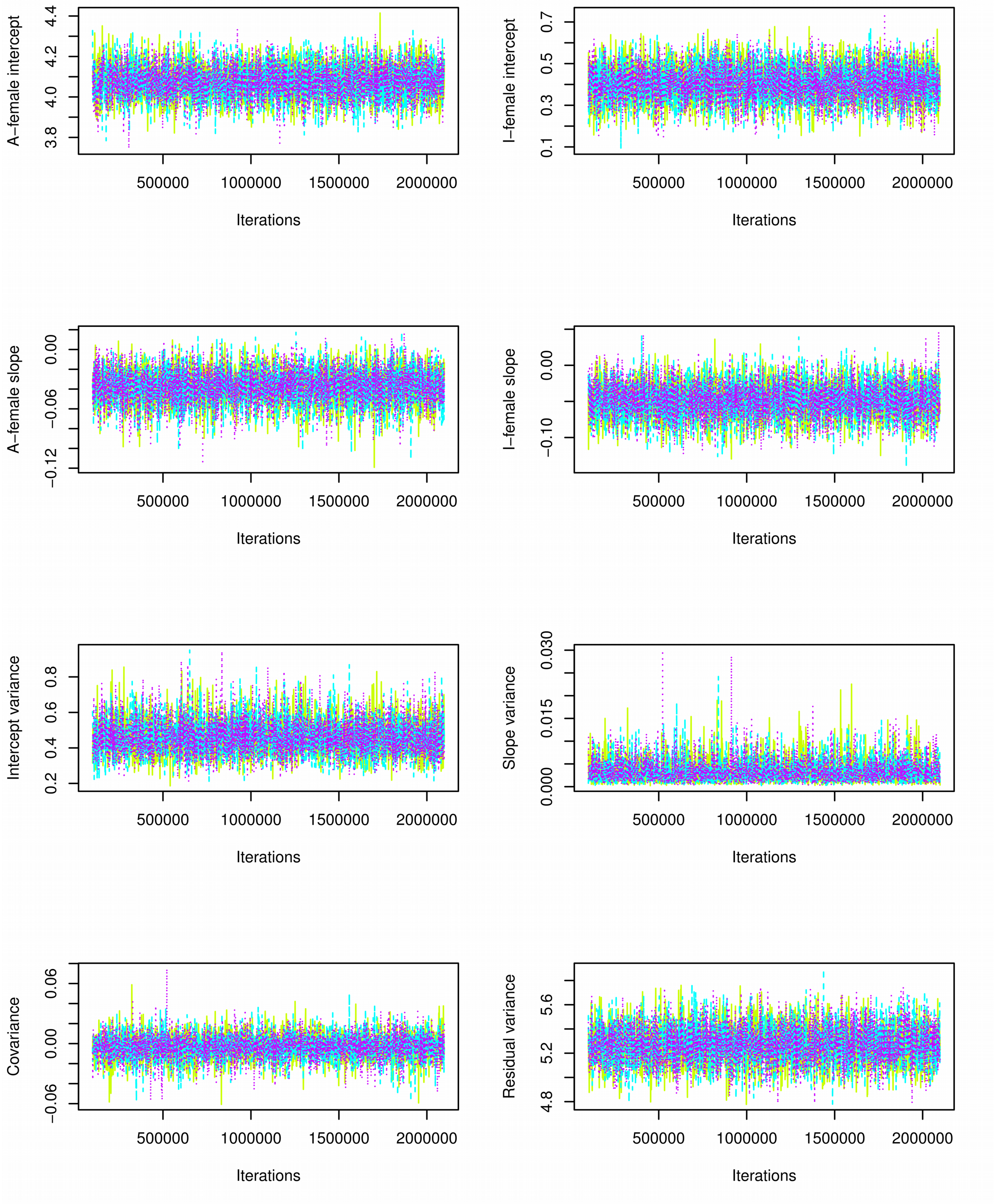

Fig. S3. MCMC trace plots for fixed-effects and variances of three Poisson glmms on the fecundity response of *I. elegans* females to water mites. Model 1 in yellow used inverse Wishart priors for

both R and G, Model 2 in turquoise used inverse Wishart priors for R parameter-expanded priors for G, Model3 in Purple used non-informative improper priors for R and parameter-expanded priors G. The fixed-effect coefficients correspond to the marginal intercepts and slopes of the fecundity response to water-mite infections. The random-effect coefficients represent the spatial and temporal variance in these parameters and the covariance between them. The response variable is the number of eggs laid by a mated female.

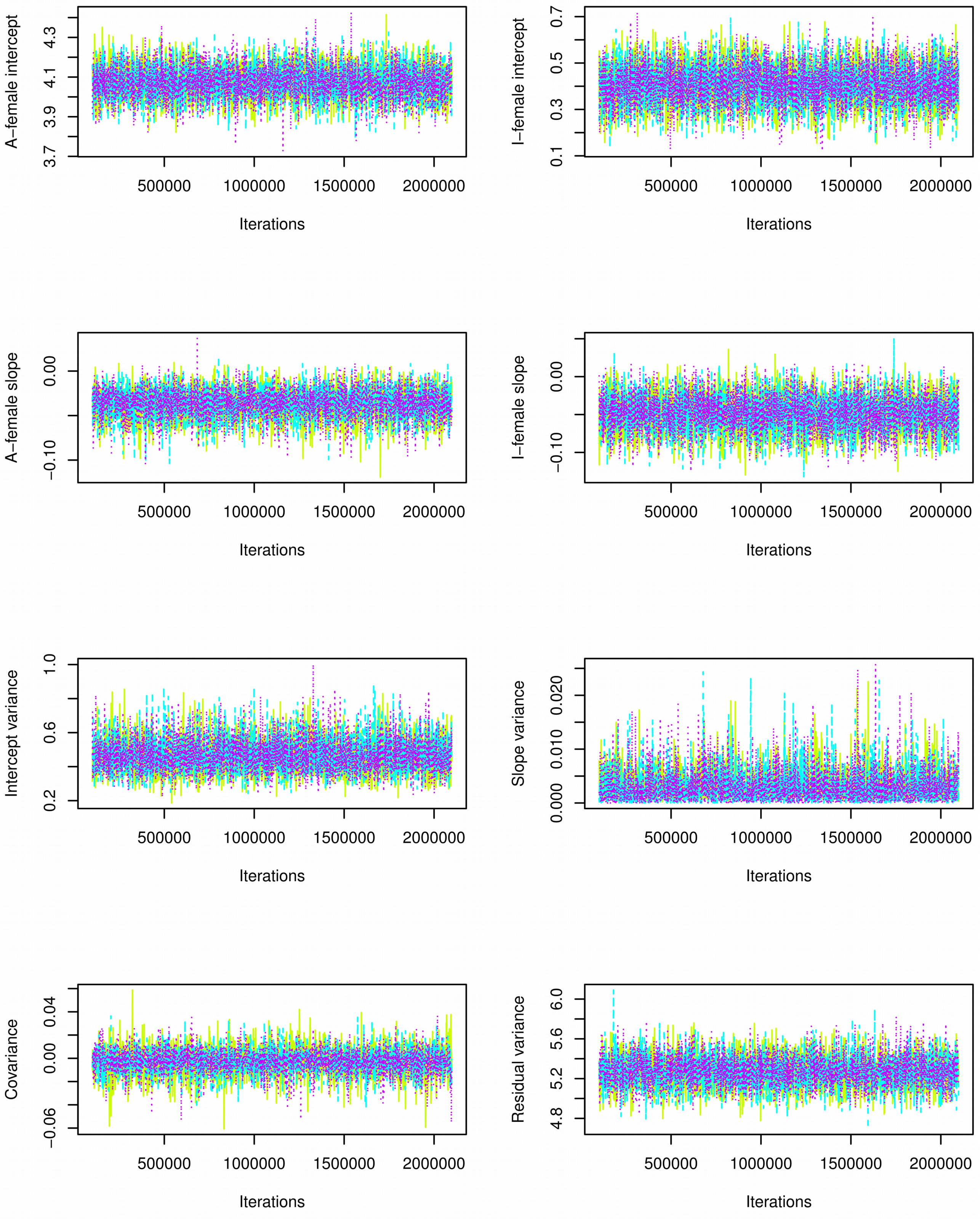

Fig. S4. MCMC trace plots for fixed-effects and variances of three independent runs of Model 1 on the fecundity response of *I. elegans* females to water mites. The fixed-effect coefficients correspond

to the marginal intercepts and slopes of the fecundity response to water-mite infections, assuming a Poisson error distribution. The random-effect coefficients represent the spatial and temporal variance in these parameters and the covariance between them. The response variable is the number of eggs laid by a mated female.

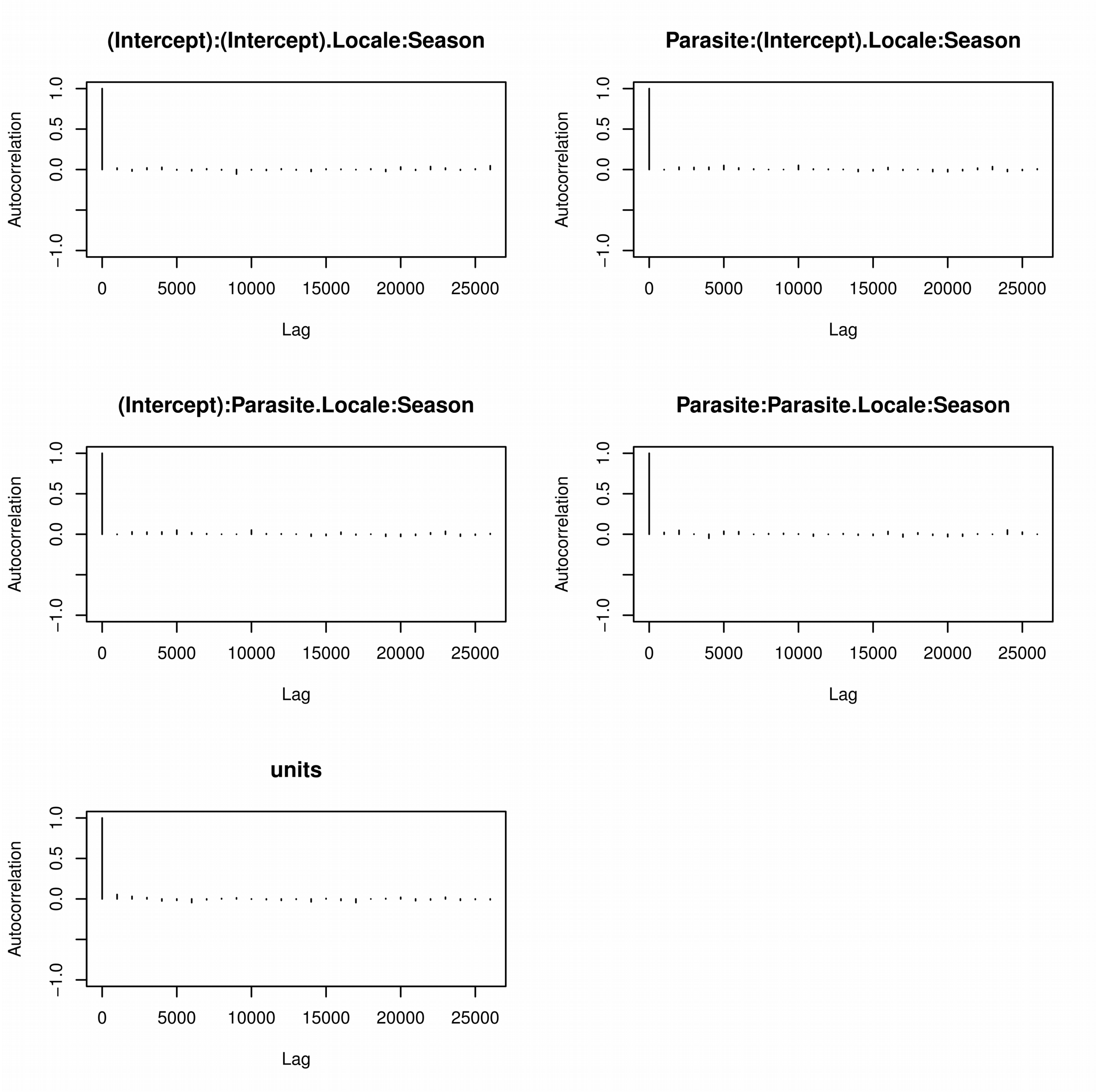

Fig. S5. Autocorrelation plot for the variance component of a Poisson MCMCglmm testing for morph-specific fecundity effects of parasitic mites on damselfly hosts.

References

1. Hadfield J. 2015 MCMCglmm Course Notes. Available at cran.us.r-project.org/web/packages/MCMCglmm/vignettes/CourseNotes.pdf Last accessed April 7, 2016.
2. Gelman A. 2006 Prior distributions for variance parameters in hierarchical models (comment on article by Browne and Draper). *Bayesian Anal.* **1**, 515-534.

Supplementary Results

Table S1. Total number of males and females of *I. elegans* sampled in this study. Females occur in three distinct and heritable morphs. 16 populations were surveyed on 5-13 seasons.

Locale	Season	A-females	I-females	O-females	Males
Fly 30 A1	2003	15	2	3	44
Fly 30 A3	2003	7	3	0	28
Genarp	2003	18	16	1	62
Gunnesbo	2003	15	2	1	44
Habo Gard	2003	16	1	5	64
Hofterup	2003	9	9	1	33
Hoje A 14	2003	29	6	1	73
Hoje A 6	2003	32	13	0	90
Hoje A 7	2003	8	7	0	32
Lomma	2003	48	23	11	120
Vallby	2003	34	12	2	89
Vombs Vattenverk	2003	36	21	3	74
Fly 30 A1	2004	14	1	1	34
Fly 30 A3	2004	36	9	6	105
Genarp	2004	38	14	4	159
Gunnesbo	2004	59	15	8	135
Habo Gard	2004	30	2	0	96
Hofterup	2004	15	5	3	39
Hoje A 14	2004	52	21	7	175
Hoje A 6	2004	32	5	4	78
Hoje A 7	2004	30	5	2	72
Lomma	2004	125	18	21	428
Vallby	2004	28	6	3	74
Vombs Vattenverk	2004	53	36	9	216
Fly 30 A1	2005	1	0	0	2
Fly 30 A3	2005	16	7	0	58
Genarp	2005	42	13	1	136
Gunnesbo	2005	27	13	2	101
Habo Gard	2005	24	4	1	81
Hofterup	2005	69	29	17	232
Hoje A 14	2005	18	3	1	124
Hoje A 6	2005	70	26	3	165
Hoje A 7	2005	13	8	1	50
Lomma	2005	142	49	11	395
Vallby	2005	24	3	2	60
Vombs Vattenverk	2005	48	44	5	185
Fly 30 A1	2006	23	3	1	47
Fly 30 A3	2006	14	8	2	53
Genarp	2006	47	6	1	160
Gunnesbo	2006	47	25	5	155

Habo Gard	2006	32	3	3	140
Hofterup	2006	80	36	15	320
Hoje A 14	2006	66	25	1	133
Hoje A 6	2006	97	37	9	217
Hoje A 7	2006	44	13	2	133
Lomma	2006	122	40	9	256
Vallby	2006	56	20	2	116
Vombs Vattenverk	2006	71	38	4	189
Fly 30 A1	2007	22	11	3	76
Fly 30 A3	2007	23	4	0	69
Genarp	2007	37	5	3	134
Gunnesbo	2007	26	13	1	74
Habo Gard	2007	49	10	6	161
Hofterup	2007	39	18	8	184
Hoje A 14	2007	60	21	3	198
Hoje A 6	2007	20	1	1	78
Hoje A 7	2007	17	4	0	82
Lomma	2007	111	42	16	284
Vallby	2007	61	9	1	92
Vombs Vattenverk	2007	119	67	8	250
Fly 30 A1	2008	1	0	0	18
Fly 30 A3	2008	8	0	1	12
Genarp	2008	56	12	7	170
Gunnesbo	2008	7	8	1	75
Habo Gard	2008	14	3	2	75
Hofterup	2008	39	15	10	142
Hoje A 14	2008	14	8	0	60
Hoje A 6	2008	32	8	0	146
Hoje A 7	2008	1	0	1	42
Lomma	2008	50	12	8	201
Vallby	2008	52	17	2	125
Vombs Vattenverk	2008	56	35	3	161
Fly 30 A1	2009	1	0	0	1
Fly 30 A3	2009	1	0	0	17
Genarp	2009	24	8	0	104
Gunnesbo	2009	6	0	0	41
Habo Gard	2009	30	11	10	152
Hofterup	2009	13	0	0	45
Hoje A 14	2009	37	10	0	113
Hoje A 6	2009	38	12	3	113
Hoje A 7	2009	0	0	0	6
Lomma	2009	41	7	9	248
Vallby	2009	51	10	1	114
Vombs Vattenverk	2009	43	28	4	157
Genarp	2010	2	0	0	1
Flackarp	2011	40	13	3	89

Fly 30 A1	2011	8	2	2	28
Fly 30 A3	2011	13	2	0	70
Genarp	2011	26	8	2	62
Gunnesbo	2011	20	6	1	91
Habo Gard	2011	12	1	2	98
Hofterup	2011	8	1	4	51
Hoje A 14	2011	13	5	2	28
Hoje A 6	2011	6	2	0	61
Hoje A 7	2011	2	0	0	9
Lomma	2011	59	1	13	152
Vallby	2011	2	0	0	3
Vombs Vattenverk	2011	58	31	8	150
Borgeby	2012	20	5	0	68
Flackarp	2012	59	12	12	201
Fly 30 A1	2012	20	1	3	77
Fly 30 A3	2012	46	12	5	227
Genarp	2012	50	15	2	232
Gunnesbo	2012	27	8	1	97
Habo Gard	2012	8	3	0	61
Hofterup	2012	11	3	0	68
Hoje A 14	2012	59	13	3	202
Hoje A 6	2012	32	3	4	160
Hoje A 7	2012	12	3	1	78
Ilstorp	2012	30	12	3	127
Lomma	2012	29	6	6	129
Lunnarp	2012	18	2	1	42
Vallby	2012	0	0	0	2
Vombs Vattenverk	2012	47	27	6	208
Borgeby	2013	13	4	1	59
Flackarp	2013	8	5	3	68
Fly 30 A1	2013	2	2	1	25
Fly 30 A3	2013	42	9	5	119
Genarp	2013	47	16	4	187
Gunnesbo	2013	23	9	2	95
Habo Gard	2013	7	2	1	61
Hoje A 14	2013	31	9	0	154
Hoje A 6	2013	10	6	4	139
Hoje A 7	2013	5	1	0	30
Ilstorp	2013	13	7	0	66
Lomma	2013	10	4	3	83
Lunnarp	2013	36	10	4	198
Vallby	2013	0	0	0	2
Vombs Vattenverk	2013	54	15	2	132
Borgeby	2014	51	20	2	143
Flackarp	2014	25	6	1	94
Fly 30 A1	2014	29	5	4	84

Fly 30 A3	2014	38	3	4	130
Genarp	2014	36	7	3	123
Gunnesbo	2014	36	15	5	114
Habo Gard	2014	12	2	1	43
Hoje A 14	2014	30	8	0	76
Hoje A 6	2014	51	15	15	209
Hoje A 7	2014	14	6	0	79
Ilstorp	2014	21	12	3	115
Lomma	2014	32	7	3	189
Lunnarp	2014	2	1	0	26
Vombs Vattenverk	2014	50	44	12	171
Borgeby	2015	67	30	7	150
Flackarp	2015	2	3	0	19
Fly 30 A1	2015	12	2	0	36
Fly 30 A3	2015	25	7	1	76
Genarp	2015	18	4	1	54
Gunnesbo	2015	25	8	1	52
Habo Gard	2015	3	1	0	21
Hoje A 14	2015	47	16	1	86
Hoje A 6	2015	16	6	0	40
Hoje A 7	2015	11	5	1	32
Ilstorp	2015	33	22	1	78
Lomma	2015	13	3	0	73
Lunnarp	2015	7	2	1	28
Vombs Vattenverk	2015	52	37	2	106
Borgeby	2016	111	19	6	296
Flackarp	2016	7	0	1	47
Fly 30 A1	2016	31	6	1	142
Fly 30 A3	2016	42	6	3	174
Genarp	2016	28	8	1	128
Gunnesbo	2016	31	10	3	118
Habo Gard	2016	12	2	1	55
Hoje A 14	2016	42	10	2	185
Hoje A 6	2016	35	4	0	155
Hoje A 7	2016	22	12	0	117
Ilstorp	2016	89	27	3	259
Lomma	2016	23	12	2	132
Lunnarp	2016	3	3	0	98
Vombs Vattenverk	2016	74	30	5	251

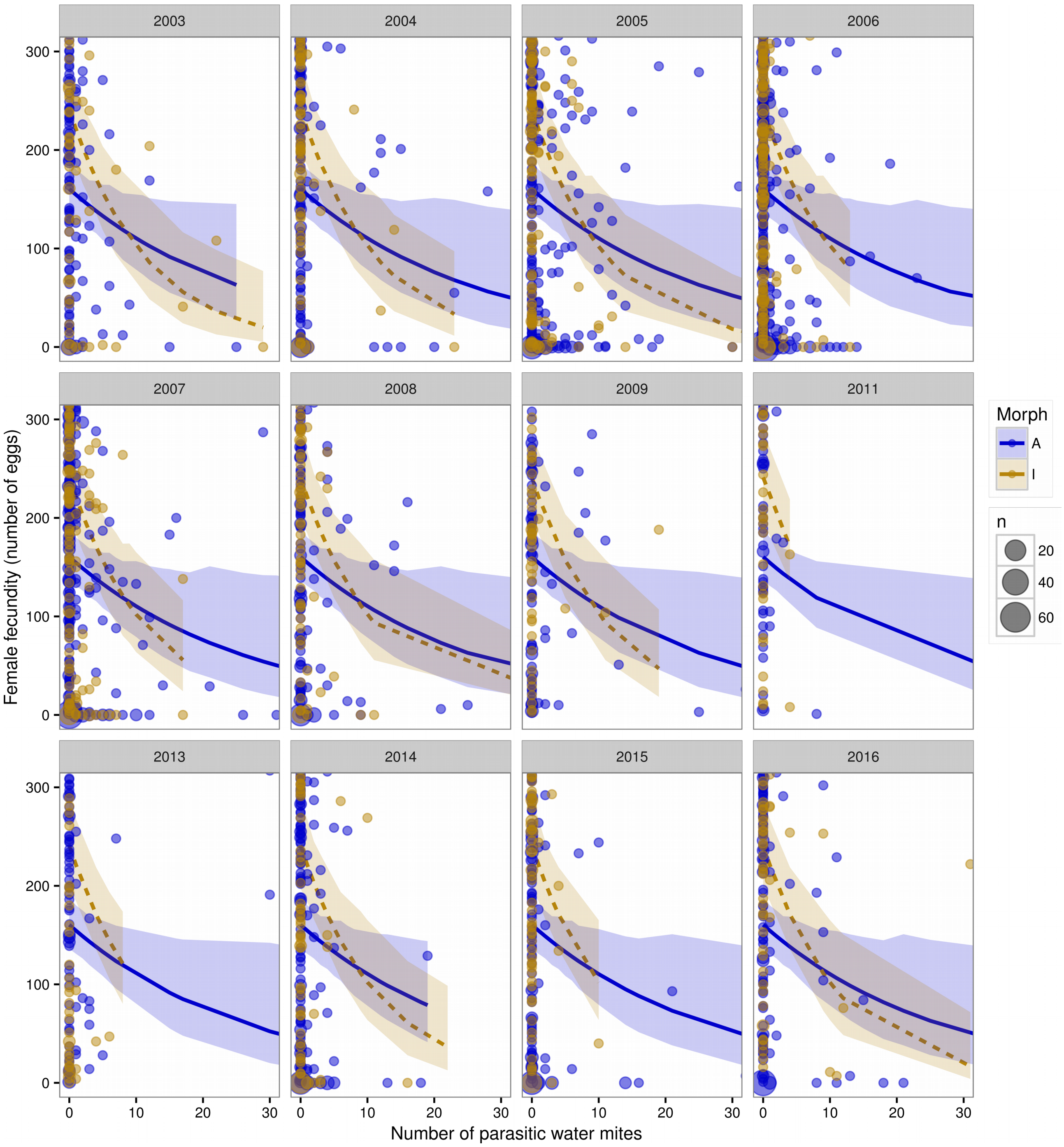

Fig. S6. Fecundity tolerance to parasitic mites among heritable colour morphs in females of *I. elegans* and by sampling season. Tolerance is here defined as a slope and a measure of the reaction norm of female fecundity to parasite load. Parasite load ranged from 0 to 56. We plot the tolerance response over more than 99% of the range of infection intensities in natural populations of *I. elegans* in southern Sweden. The fitted lines represent the predictions of the effects of water mites on female fecundity and the shaded areas cover the 95% credible intervals. The circles represent

individual females and the size of the circles is proportional to to the number of females with the same number or parasites and fecundity.

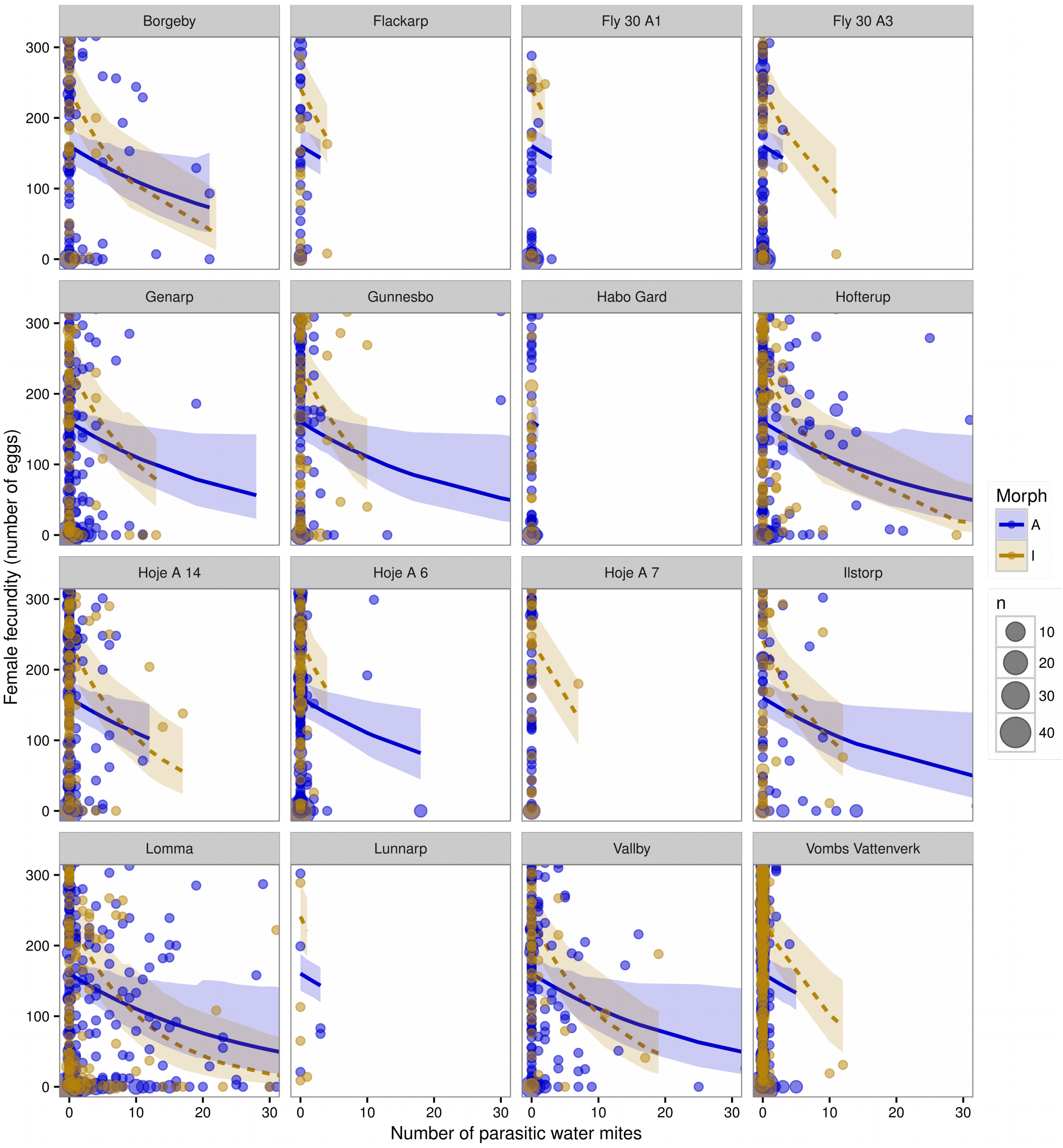

Fig. S7. Fecundity tolerance to parasitic mites among heritable colour morphs in females of *I. elegans* and by sampling season. Tolerance is here defined as a slope and a measure of the reaction norm of female fecundity to parasite load. Parasite load ranged from 0 to 56. We plot the tolerance response over more than 99% of the range of infection intensities in natural populations of *I. elegans* in southern Sweden. The fitted lines represent the predictions of the effects of water mites on female fecundity and the shaded areas cover the 95% credible intervals. The circles represent

individual females and the size of the circles is proportional to to the number of females with the same number or parasites and fecundity.

Population variance in resistance

Table S2. Population effects on the posterior mean estimates of parasite prevalence (logit scale) and infection intensity (log scale) in *I. elegans* damselflies. We report as P values the proportion of the posterior distributions in which the population estimates overlap with the global intercept.

Locale	Season	Prevalence	Lower 95% CI	Upper 95% CI	pMCMC	Intensity	Lower 95% CI	Upper 95% CI	pMCMC
Fly 30 A1	2003	-0.65	-1.77	0.4	0.24	-0.94	-2.32	0.51	0.18
Fly 30 A3	2003	-0.15	-1.39	0.88	0.81	-0.92	-2.26	0.52	0.18
Genarp	2003	-0.59	-1.44	0.29	0.18	-0.59	-1.7	0.53	0.31
Gunnesbo	2003	0.22	-0.67	0.99	0.55	-0.71	-1.75	0.35	0.18
Habo Gard	2003	-0.65	-1.67	0.3	0.18	0.35	-0.86	1.4	0.55
Hofterup	2003	0.98	0.26	1.66	**0.01**	0.86	0.12	1.59	**0.02**
Hoje A 14	2003	1.37	0.89	1.86	**< 0.01**	0.69	0.19	1.16	**0.01**
Hoje A 6	2003	-0.21	-0.94	0.4	0.55	-0.64	-1.52	0.31	0.16
Hoje A 7	2003	-1.13	-2.59	0.15	0.07	0.51	-0.97	1.82	0.48
Lomma	2003	1.76	1.41	2.13	**< 0.01**	1.34	0.99	1.66	**< 0.01**
Vallby	2003	0.72	0.23	1.24	**< 0.01**	-0.22	-0.82	0.38	0.48
Vombs V	2003	0.05	-0.55	0.71	0.85	-1.37	-2.28	-0.32	**< 0.01**
Fly 30 A1	2004	-1.87	-3.58	-0.31	**0.01**	0	-1.72	1.84	1
Fly 30 A3	2004	-1.76	-2.88	-0.65	**< 0.01**	-0.67	-2.15	0.78	0.36
Genarp	2004	-1.49	-2.45	-0.63	**< 0.01**	-0.56	-1.98	0.58	0.39
Gunnesbo	2004	-1.22	-2.07	-0.47	**< 0.01**	-0.11	-1.05	0.87	0.82
Habo Gard	2004	-1.61	-2.8	-0.54	**< 0.01**	-0.25	-1.59	1.18	0.73
Hofterup	2004	1.57	0.94	2.11	**< 0.01**	1.01	0.48	1.57	**< 0.01**
Hoje A 14	2004	-1.05	-1.73	-0.34	**< 0.01**	-0.37	-1.26	0.6	0.44
Hoje A 6	2004	-0.8	-1.66	0.07	**0.05**	-1.25	-2.6	-0.02	**0.04**
Hoje A 7	2004	-2.43	-4.15	-0.91	**< 0.01**	-0.01	-1.67	1.82	1
Lomma	2004	2.37	2.1	2.65	**< 0.01**	1.85	1.64	2.1	**< 0.01**
Vallby	2004	-0.93	-1.87	-0.01	**0.04**	0.23	-0.86	1.32	0.66
Vombs V	2004	-1.24	-2	-0.61	**< 0.01**	-1.21	-2.39	-0.07	**0.04**
Fly 30 A1	2005	-0.51	-3.07	1.52	0.7	0.01	-1.65	1.71	0.98
Fly 30 A3	2005	-0.86	-1.91	0.21	0.09	-0.68	-1.95	0.69	0.33
Genarp	2005	1.2	0.78	1.57	**< 0.01**	-0.55	-1.04	-0.04	**0.03**
Gunnesbo	2005	0.29	-0.3	0.84	0.31	-0.72	-1.53	-0.01	**0.05**
Habo Gard	2005	-0.9	-1.77	0.07	**0.05**	-1.1	-2.36	0.45	0.13
Hofterup	2005	2.6	2.3	2.91	**< 0.01**	0.92	0.68	1.17	**< 0.01**
Hoje A 14	2005	0.26	-0.32	0.88	0.38	0.59	-0.06	1.18	0.07
Hoje A 6	2005	-0.37	-0.89	0.19	0.19	-0.92	-1.72	-0.14	**0.02**
Hoje A 7	2005	-0.74	-1.89	0.28	0.17	-0.63	-1.97	0.59	0.35
Lomma	2005	2.3	2.02	2.56	**< 0.01**	1.1	0.89	1.35	**< 0.01**
Vallby	2005	0.53	-0.12	1.13	0.12	0.23	-0.45	0.98	0.53

Vombs V	2005	-0.26	-0.81	0.22	0.33	0.63	-0.04	1.18	**0.05**
Fly 30 A1	2006	-0.57	-1.62	0.35	0.26	-0.33	-1.46	0.87	0.59
Fly 30 A3	2006	-1.14	-2.42	0.05	**0.05**	-0.09	-1.39	1.36	0.89
Genarp	2006	0.89	0.46	1.3	**< 0.01**	0.17	-0.29	0.61	0.43
Gunnesbo	2006	0.57	0.1	0.98	**< 0.01**	0.24	-0.25	0.7	0.33
Habo Gard	2006	-0.93	-1.75	-0.1	**< 0.01**	-0.42	-1.46	0.59	0.43
Hofterup	2006	0.6	0.26	0.95	**< 0.01**	0.2	-0.19	0.58	0.29
Hoje A 14	2006	-0.15	-0.72	0.35	0.58	-0.54	-1.33	0.15	0.14
Hoje A 6	2006	-1.87	-2.66	-1.04	**< 0.01**	0.32	-0.61	1.34	0.54
Hoje A 7	2006	-2.31	-3.6	-1.05	**< 0.01**	-0.43	-2.08	1.12	0.6
Lomma	2006	1.6	1.28	1.9	**< 0.01**	0.67	0.38	0.94	**< 0.01**
Vallby	2006	0.22	-0.28	0.71	0.38	0.33	-0.23	0.9	0.26
Vombs V	2006	-1.38	-2.13	-0.66	**< 0.01**	-0.41	-1.4	0.62	0.43
Fly 30 A1	2007	0.01	-0.67	0.69	0.97	-0.74	-1.56	0.22	0.09
Fly 30 A3	2007	0.05	-0.71	0.77	0.86	-0.76	-1.77	0.16	0.13
Genarp	2007	0.82	0.37	1.24	**< 0.01**	-0.04	-0.63	0.46	0.87
Gunnesbo	2007	0.52	-0.1	1.05	0.1	-0.64	-1.39	0.16	0.11
Habo Gard	2007	-0.23	-0.78	0.32	0.44	-0.97	-1.79	-0.11	**0.02**
Hofterup	2007	1.94	1.61	2.3	**< 0.01**	0.19	-0.15	0.51	0.3
Hoje A 14	2007	0.11	-0.38	0.53	0.63	-0.25	-0.82	0.32	0.39
Hoje A 6	2007	-0.39	-1.27	0.48	0.39	0.44	-0.48	1.41	0.38
Hoje A 7	2007	-1.76	-3.09	-0.44	**< 0.01**	-0.41	-2.11	1.14	0.64
Lomma	2007	2.39	2.12	2.68	**< 0.01**	0.89	0.66	1.16	**< 0.01**
Vallby	2007	0.97	0.56	1.4	**< 0.01**	-0.35	-0.85	0.21	0.21
Vombs V	2007	-1.37	-1.99	-0.77	**< 0.01**	-1.42	-2.43	-0.33	**0.01**
Fly 30 A1	2008	0.6	-0.62	1.82	0.34	-0.36	-1.65	0.91	0.57
Fly 30 A3	2008	-1.44	-3.36	0.29	0.1	-0.03	-1.86	1.7	0.95
Genarp	2008	0.43	-0.02	0.89	0.07	-0.21	-0.77	0.29	0.43
Gunnesbo	2008	0.21	-0.47	0.97	0.58	-0.66	-1.67	0.3	0.17
Habo Gard	2008	-1.68	-3.09	-0.43	**< 0.01**	-0.39	-2.13	1.07	0.65
Hofterup	2008	2.1	1.76	2.46	**< 0.01**	1.1	0.77	1.41	**< 0.01**
Hoje A 14	2008	-0.85	-2	0.18	0.1	-0.91	-2.43	0.55	0.24
Hoje A 6	2008	-2.24	-3.64	-1.09	**< 0.01**	-0.43	-1.96	1.05	0.59
Hoje A 7	2008	-1.71	-3.44	0	**0.03**	0.03	-1.53	1.93	0.98
Lomma	2008	1.77	1.45	2.12	**< 0.01**	0.66	0.35	1.01	**< 0.01**
Vallby	2008	0.57	0.1	0.99	**0.02**	0.26	-0.24	0.78	0.31
Vombs V	2008	-1.2	-1.95	-0.46	**< 0.01**	-1.44	-2.8	-0.17	**0.02**
Fly 30 A1	2009	-0.4	-2.66	1.91	0.77	-0.03	-1.77	1.79	0.97
Fly 30 A3	2009	-1.19	-3.19	0.63	0.21	-0.02	-1.59	1.79	0.99
Genarp	2009	0.29	-0.35	0.85	0.33	0.59	-0.01	1.28	0.07
Gunnesbo	2009	0.95	0.18	1.66	**0.02**	0.09	-0.74	0.96	0.84
Habo Gard	2009	-0.79	-1.5	-0.09	**0.01**	-0.48	-1.5	0.5	0.34
Hofterup	2009	1.93	1.35	2.45	**< 0.01**	1.06	0.49	1.57	**< 0.01**
Hoje A 14	2009	-2.16	-3.47	-0.93	**< 0.01**	-0.4	-2.05	1.26	0.64
Hoje A 6	2009	-1.84	-2.91	-0.79	**< 0.01**	-0.71	-2.17	0.63	0.34
Hoje A 7	2009	-0.71	-3.08	1.32	0.59	0.01	-1.85	1.72	0.99
Lomma	2009	1.8	1.47	2.1	**< 0.01**	1.34	1.07	1.67	**< 0.01**
Vallby	2009	1.51	1.12	1.95	**< 0.01**	0.33	-0.05	0.77	0.12
Vombs V	2009	-1.82	-2.78	-0.84	**< 0.01**	-0.67	-2.02	0.58	0.33

Genarp	2010	2.52	0.8	4.45	**0.01**	0.08	-1.13	1.28	0.91
Lomma	2010	1.33	-0.85	3.82	0.29	-0.05	-1.59	1.42	0.93
Flackarp	2011	-0.12	-0.73	0.49	0.72	-0.54	-1.33	0.31	0.2
Fly 30 A1	2011	0.55	-0.34	1.4	0.23	-0.22	-1.22	0.76	0.68
Fly 30 A3	2011	-2.19	-3.87	-0.62	**< 0.01**	0.03	-1.65	1.75	0.98
Genarp	2011	0.33	-0.34	0.97	0.33	0.79	0.12	1.51	**0.03**
Gunnesbo	2011	1.8	1.36	2.23	**< 0.01**	1.6	1.19	2.03	**< 0.01**
Habo Gard	2011	-1.4	-2.62	-0.35	**0.01**	-0.26	-1.63	1.13	0.74
Hofterup	2011	1.7	1.12	2.29	**< 0.01**	1.38	0.84	1.93	**< 0.01**
Hoje A 14	2011	-0.46	-1.58	0.58	0.42	-0.05	-1.32	1.11	0.94
Hoje A 6	2011	-1	-2.24	0.21	0.1	0.36	-0.94	1.74	0.6
Hoje A 7	2011	-1.01	-3.14	0.85	0.33	-0.03	-1.81	1.63	0.97
Lomma	2011	0.54	0.1	0.96	**0.02**	0.52	0.03	0.98	**0.03**
Vallby	2011	0.36	-1.57	2.16	0.68	0.21	-1.2	1.72	0.79
Vombs V	2011	-1.06	-1.74	-0.33	**< 0.01**	-0.81	-1.76	0.18	0.11
Borgeby	2012	-0.53	-1.47	0.35	0.24	-0.17	-1.23	0.89	0.77
Flackarp	2012	0.4	0	0.84	0.06	0.04	-0.44	0.54	0.89
Fly 30 A1	2012	-1.77	-3.14	-0.52	**< 0.01**	-0.47	-2	1.11	0.55
Fly 30 A3	2012	-0.57	-1.22	-0.01	**0.05**	-0.87	-1.66	0.04	**0.05**
Genarp	2012	0.66	0.26	1.06	**< 0.01**	0.11	-0.32	0.59	0.63
Gunnesbo	2012	2.9	2.5	3.3	**< 0.01**	2.17	1.86	2.48	**< 0.01**
Habo Gard	2012	0	-0.88	0.83	0.96	0.32	-0.68	1.27	0.52
Hofterup	2012	1.99	1.5	2.47	**< 0.01**	0.95	0.5	1.43	**< 0.01**
Hoje A 14	2012	-1.98	-2.93	-0.99	**< 0.01**	-0.33	-1.58	1.06	0.62
Hoje A 6	2012	-0.18	-0.77	0.42	0.59	-0.11	-0.86	0.6	0.79
Hoje A 7	2012	-2.23	-3.97	-0.72	**< 0.01**	0.01	-1.76	1.69	0.97
Ilstorp	2012	1.85	1.46	2.22	**< 0.01**	0.27	-0.11	0.66	0.16
Lomma	2012	2.03	1.66	2.41	**< 0.01**	1.68	1.33	2.02	**< 0.01**
Lunnarp	2012	-2.03	-3.66	-0.54	**< 0.01**	-0.05	-1.93	1.6	0.97
Vallby	2012	-0.35	-2.86	2.11	0.83	0.03	-1.73	1.74	0.98
Vombs V	2012	-0.11	-0.61	0.38	0.65	-0.26	-0.92	0.35	0.43
Borgeby	2013	-0.53	-1.56	0.41	0.29	-0.34	-1.48	0.76	0.59
Flackarp	2013	0.81	0.2	1.5	**0.02**	-0.94	-1.76	-0.08	0.02
Fly 30 A1	2013	-1.49	-3.45	0.2	0.09	0.01	-1.76	1.82	0.99
Fly 30 A3	2013	-1.36	-2.31	-0.42	**< 0.01**	-0.5	-1.69	0.76	0.44
Genarp	2013	0.72	0.33	1.16	**< 0.01**	-0.45	-0.92	0.04	0.08
Gunnesbo	2013	2.65	2.24	3.05	**< 0.01**	1.71	1.38	2.04	**< 0.01**
Habo Gard	2013	0.35	-0.39	1.12	0.37	0.56	-0.32	1.4	0.2
Hoje A 14	2013	-0.16	-0.75	0.44	0.61	-0.5	-1.31	0.27	0.21
Hoje A 6	2013	-0.23	-0.9	0.44	0.5	-0.38	-1.28	0.4	0.39
Hoje A 7	2013	-1.62	-3.46	0.05	**0.05**	0.03	-1.68	1.67	0.98
Ilstorp	2013	1.73	1.23	2.24	**< 0.01**	0.22	-0.28	0.74	0.41
Lomma	2013	2.18	1.75	2.67	**< 0.01**	1.12	0.71	1.55	**< 0.01**
Lunnarp	2013	0.18	-0.31	0.66	0.47	-0.53	-1.12	0.09	0.09
Vallby	2013	-0.32	-2.99	1.92	0.85	0.01	-1.78	1.73	1
Vombs V	2013	-0.37	-1.01	0.19	0.21	-1.01	-1.85	-0.06	**0.03**
Borgeby	2014	2.1	1.76	2.46	**< 0.01**	0.66	0.35	0.98	**< 0.01**
Flackarp	2014	-0.1	-0.75	0.57	0.8	-0.65	-1.58	0.21	0.14
Fly 30 A1	2014	-0.21	-0.88	0.5	0.56	0.4	-0.38	1.18	0.32

Fly 30 A3	2014	0.2	-0.32	0.73	0.47	-0.16	-0.83	0.53	0.67
Genarp	2014	0.66	0.21	1.14	**0.01**	-1.02	-1.76	-0.39	**< 0.01**
Gunnesbo	2014	2.23	1.88	2.62	**< 0.01**	0.87	0.54	1.2	**< 0.01**
Habo Gard	2014	-0.53	-1.58	0.54	0.34	-0.44	-1.78	0.8	0.5
Hoje A 14	2014	-0.31	-1.07	0.44	0.44	-0.1	-0.95	0.91	0.84
Hoje A 6	2014	-2.25	-3.39	-1.28	**< 0.01**	0.78	-0.37	2.1	0.22
Hoje A 7	2014	-1	-2.06	-0.02	**0.03**	0.55	-0.58	1.66	0.34
Ilstorp	2014	2.03	1.63	2.4	**< 0.01**	0.55	0.17	0.95	**0.01**
Lomma	2014	2.71	2.39	3.05	**< 0.01**	1.69	1.42	1.96	**< 0.01**
Lunnarp	2014	-0.19	-1.52	1.08	0.81	0.02	-1.39	1.26	0.97
Vombs V	2014	0.7	0.33	1.13	**< 0.01**	-0.41	-0.88	0.05	0.1
Borgeby	2015	0.54	0.11	0.93	**0.01**	0.41	-0.04	0.88	0.08
Flackarp	2015	0.33	-0.9	1.55	0.59	0.53	-0.67	1.54	0.35
Fly 30 A1	2015	-0.17	-1.14	0.86	0.79	-0.35	-1.6	0.75	0.59
Fly 30 A3	2015	-0.35	-1.16	0.43	0.4	-0.04	-0.93	0.9	0.94
Genarp	2015	0	-0.79	0.78	0.97	-1.19	-2.44	-0.08	**0.05**
Gunnesbo	2015	1.22	0.66	1.73	**< 0.01**	0.9	0.32	1.45	**< 0.01**
Habo Gard	2015	-0.69	-2.15	0.86	0.4	0.23	-1.21	1.62	0.76
Hoje A 14	2015	-1.27	-2.28	-0.38	**< 0.01**	-0.28	-1.55	0.8	0.64
Hoje A 6	2015	0.05	-0.81	0.9	0.9	0.29	-0.71	1.2	0.54
Hoje A 7	2015	-0.12	-1.11	0.85	0.87	-0.4	-1.64	0.79	0.51
Ilstorp	2015	1.33	0.89	1.75	**< 0.01**	0.8	0.38	1.26	**< 0.01**
Lomma	2015	2.19	1.71	2.63	**< 0.01**	1.45	1.01	1.88	**< 0.01**
Lunnarp	2015	-0.13	-1.27	0.95	0.88	-0.64	-1.99	0.63	0.35
Vombs V	2015	-0.99	-1.76	-0.26	**0.01**	-1.03	-2.21	0.11	0.08
Borgeby	2016	0.85	0.52	1.2	**< 0.01**	0.58	0.26	0.93	**< 0.01**
Flackarp	2016	-0.14	-1.15	0.83	0.8	-0.05	-1.2	1.06	0.94
Fly 30 A1	2016	-0.8	-1.53	0	**0.04**	-0.17	-1.16	0.75	0.73
Fly 30 A3	2016	-0.58	-1.21	0.08	0.07	0.46	-0.28	1.18	0.23
Genarp	2016	-0.15	-0.76	0.47	0.64	-0.9	-1.75	0.04	**0.05**
Gunnesbo	2016	1.1	0.65	1.52	**< 0.01**	0.1	-0.4	0.55	0.7
Habo Gard	2016	-1.46	-2.77	-0.05	0.02	-0.44	-2.09	1.19	0.57
Hoje A 14	2016	-0.83	-1.55	-0.18	0.01	-1.09	-2.07	0	**0.02**
Hoje A 6	2016	1.01	0.56	1.43	**< 0.01**	0.04	-0.46	0.51	0.87
Hoje A 7	2016	-1.38	-2.44	-0.36	**< 0.01**	0.71	-0.49	1.74	0.22
Ilstorp	2016	2.05	1.75	2.35	**< 0.01**	0.97	0.72	1.24	**< 0.01**
Lomma	2016	2.28	1.91	2.62	**< 0.01**	1.47	1.16	1.8	**< 0.01**
Lunnarp	2016	-0.93	-1.98	0.09	0.06	-0.63	-1.88	0.66	0.35
Vombs V	2016	-0.64	-1.2	-0.13	**0.01**	-0.48	-1.22	0.19	0.19

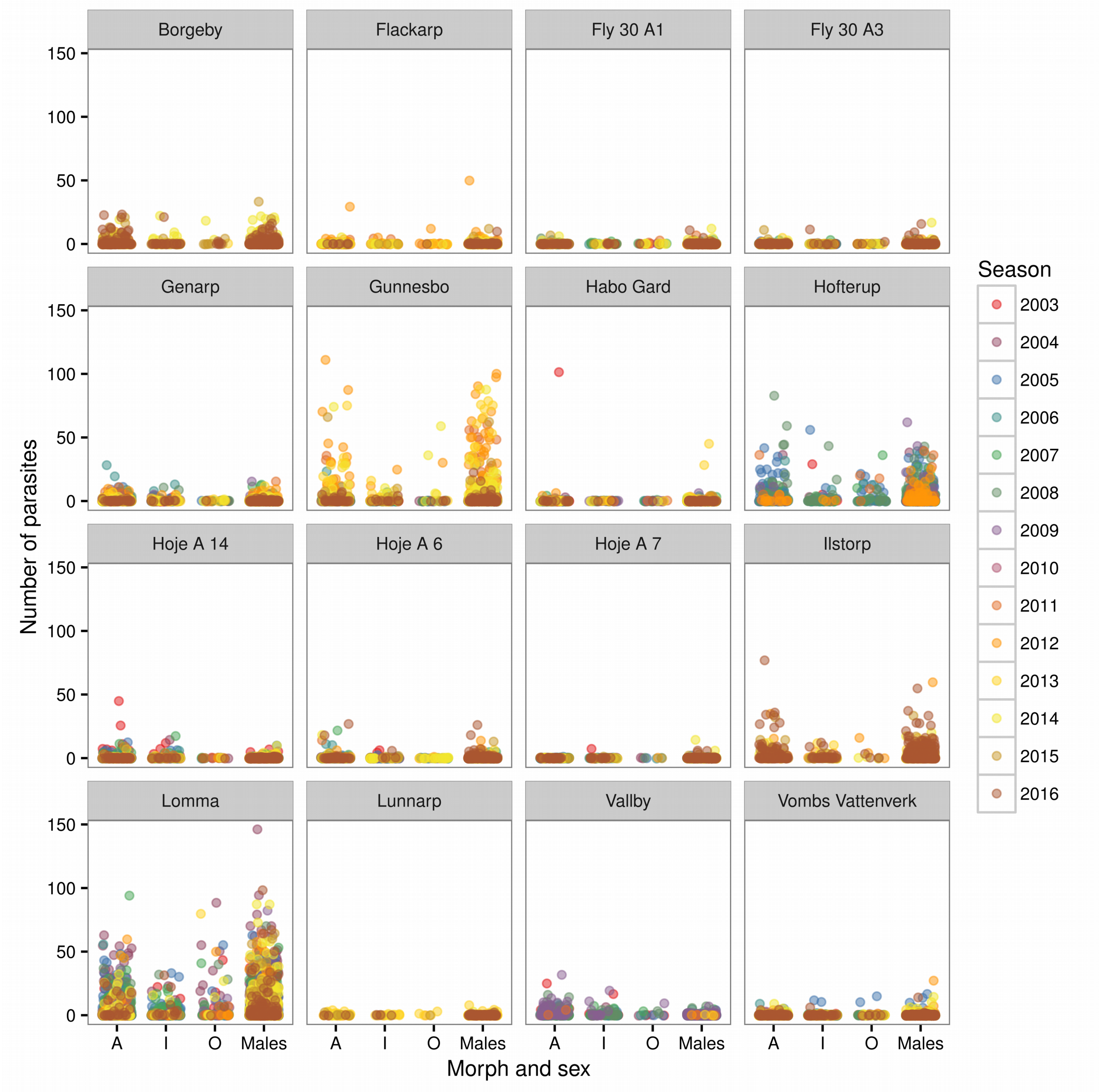

Fig. S8. Water mite infections across 16 populations of *I. elegans* damselflies and up to 14 years of field sampling. Points represent individual water mite counts.

Population variance in female fecundity

Table S2. Population effects on the posterior mean estimates of the intercept of the reaction norm of female fecundity to water mite infections in *I. elegans* females. Coefficients are in log scale. We report as P values the proportion of the posterior distributions in which the population estimates overlap with the global intercept.

Locale	Season	Fecundity intercept	Lower 95% CI	Upper 95% CI	pMCMC
Fly 30 A1	2003	0.14	-0.87	1.18	0.81
Fly 30 A3	2003	0.00	-1.12	1.09	1.00
Genarp	2003	0.40	-0.40	1.07	0.29
Gunnesbo	2003	0.80	-0.11	1.72	0.09
Habo Gard	2003	0.00	-1.01	1.01	0.99
Hofterup	2003	-0.36	-1.23	0.56	0.44
Hoje A 14	2003	0.50	-0.40	1.39	0.27
Hoje A 6	2003	0.63	-0.06	1.38	0.10
Hoje A 7	2003	0.30	-0.66	1.18	0.52
Lomma	2003	-0.08	-0.80	0.58	0.80
Vallby	2003	-0.29	-1.09	0.42	0.42
Vombs V	2003	0.73	0.16	1.38	**0.02**
Fly 30 A1	2004	0.06	-1.11	1.07	0.88
Fly 30 A3	2004	-0.39	-1.26	0.51	0.39
Genarp	2004	-0.34	-1.02	0.40	0.35
Gunnesbo	2004	0.16	-0.50	0.86	0.66
Habo Gard	2004	0.24	-1.00	1.53	0.68
Hofterup	2004	-0.14	-1.04	0.80	0.80
Hoje A 14	2004	0.54	-0.04	1.16	0.09
Hoje A 6	2004	-0.81	-1.83	0.22	0.11
Hoje A 7	2004	0.15	-0.74	1.04	0.74
Lomma	2004	-0.36	-1.06	0.27	0.30
Vallby	2004	0.72	-0.08	1.65	0.09
Vombs V	2004	0.39	-0.14	0.93	0.17
Fly 30 A3	2005	0.52	-0.35	1.35	0.24
Genarp	2005	-1.10	-1.82	-0.44	**< 0.01**
Gunnesbo	2005	0.68	-0.03	1.46	0.07
Habo Gard	2005	-0.78	-1.82	0.39	0.17
Hofterup	2005	0.16	-0.37	0.72	0.57
Hoje A 14	2005	0.08	-0.82	1.03	0.85
Hoje A 6	2005	0.02	-0.46	0.56	0.91
Hoje A 7	2005	0.09	-0.80	0.93	0.88
Lomma	2005	-0.71	-1.18	-0.25	**< 0.01**
Vallby	2005	0.14	-0.84	1.10	0.78
Vombs V	2005	0.11	-0.37	0.66	0.65
Fly 30 A1	2006	-1.70	-2.56	-0.77	**< 0.01**

Fly 30 A3	2006	-0.75	-1.64	0.10	0.08
Genarp	2006	-0.28	-0.93	0.50	0.44
Gunnesbo	2006	0.29	-0.26	0.84	0.30
Habo Gard	2006	-0.76	-1.85	0.21	0.13
Hofterup	2006	-0.16	-0.64	0.32	0.52
Hoje A 14	2006	0.30	-0.16	0.79	0.24
Hoje A 6	2006	-0.35	-0.81	0.09	0.12
Hoje A 7	2006	0.53	-0.09	1.18	0.10
Lomma	2006	-1.35	-1.85	-0.91	**< 0.01**
Vallby	2006	-0.23	-0.79	0.33	0.43
Vombs V	2006	0.16	-0.31	0.71	0.52
Fly 30 A1	2007	-0.82	-1.52	-0.01	0.03
Fly 30 A3	2007	-0.24	-1.02	0.61	0.56
Genarp	2007	-0.93	-1.79	-0.17	**0.03**
Gunnesbo	2007	0.39	-0.42	1.19	0.33
Habo Gard	2007	-0.42	-1.22	0.29	0.25
Hofterup	2007	-0.33	-0.96	0.27	0.26
Hoje A 14	2007	0.09	-0.46	0.65	0.75
Hoje A 6	2007	0.37	-0.60	1.30	0.43
Hoje A 7	2007	0.26	-0.74	1.34	0.61
Lomma	2007	-0.86	-1.37	-0.39	**< 0.01**
Vallby	2007	0.17	-0.40	0.74	0.56
Vombs V	2007	0.19	-0.23	0.68	0.41
Fly 30 A3	2008	-0.32	-1.53	0.82	0.59
Genarp	2008	-1.05	-1.70	-0.36	**< 0.01**
Gunnesbo	2008	0.02	-1.36	1.18	0.98
Habo Gard	2008	0.36	-0.75	1.37	0.50
Hofterup	2008	-0.06	-0.77	0.71	0.89
Hoje A 14	2008	0.04	-1.08	1.09	0.95
Hoje A 6	2008	0.01	-0.70	0.80	1.00
Hoje A 7	2008	-0.37	-1.55	0.98	0.57
Lomma	2008	-1.47	-2.37	-0.72	**< 0.01**
Vallby	2008	0.11	-0.57	0.88	0.75
Vombs V	2008	0.12	-0.47	0.66	0.66
Fly 30 A3	2009	0.04	-1.20	1.29	0.95
Genarp	2009	0.08	-0.78	0.90	0.86
Gunnesbo	2009	0.10	-1.14	1.49	0.87
Habo Gard	2009	-0.05	-1.00	0.98	0.93
Hofterup	2009	0.12	-1.22	1.44	0.88
Hoje A 14	2009	0.32	-0.44	1.10	0.43
Hoje A 6	2009	0.63	-0.15	1.41	0.12
Lomma	2009	-0.24	-1.16	0.74	0.63
Vallby	2009	0.45	-0.30	1.21	0.26
Vombs V	2009	0.52	-0.07	1.18	0.11
Flackarp	2011	0.30	-0.46	1.01	0.41
Genarp	2011	0.14	-0.94	1.25	0.82

Gunnesbo	2011	0.44	-0.54	1.56	0.41
Habo Gard	2011	0.11	-1.20	1.35	0.86
Hofterup	2011	0.15	-1.19	1.36	0.81
Hoje A 14	2011	0.17	-1.08	1.37	0.78
Lomma	2011	0.25	-0.75	1.28	0.62
Vombs V	2011	0.83	0.20	1.43	**0.01**
Borgeby	2013	0.69	-0.22	1.65	0.15
Flackarp	2013	0.36	-0.81	1.45	0.54
Fly 30 A1	2013	0.10	-1.14	1.37	0.87
Fly 30 A3	2013	0.37	-0.32	1.11	0.31
Genarp	2013	0.50	-0.22	1.15	0.14
Gunnesbo	2013	0.60	-0.35	1.54	0.23
Habo Gard	2013	0.00	-1.17	1.21	1.00
Hoje A 14	2013	0.57	-0.11	1.31	0.11
Hoje A 6	2013	0.38	-0.63	1.38	0.47
Hoje A 7	2013	0.48	-0.66	1.61	0.38
Ilstorp	2013	0.38	-0.58	1.39	0.45
Lunnarp	2013	0.87	0.02	1.61	**0.04**
Vombs V	2013	0.57	-0.15	1.23	0.11
Borgeby	2014	-0.01	-0.60	0.61	0.97
Flackarp	2014	-0.22	-1.04	0.58	0.59
Fly 30 A1	2014	0.17	-0.80	1.22	0.74
Fly 30 A3	2014	0.09	-0.80	0.92	0.87
Genarp	2014	-0.03	-0.84	0.77	0.96
Gunnesbo	2014	0.14	-0.58	0.92	0.73
Habo Gard	2014	-0.08	-1.16	1.13	0.90
Hoje A 14	2014	-0.90	-1.64	-0.12	**0.03**
Hoje A 6	2014	-0.44	-1.01	0.18	0.16
Hoje A 7	2014	0.04	-1.17	1.19	0.92
Ilstorp	2014	0.11	-0.66	0.84	0.75
Lomma	2014	-0.03	-0.90	0.81	0.96
Lunnarp	2014	-0.18	-1.44	1.14	0.79
Vombs V	2014	0.19	-0.24	0.72	0.46
Borgeby	2015	0.48	-0.06	0.93	0.07
Fly 30 A1	2015	-0.76	-2.11	0.50	0.25
Fly 30 A3	2015	-1.44	-2.38	-0.45	**< 0.01**
Genarp	2015	-0.49	-1.51	0.56	0.35
Gunnesbo	2015	-0.12	-1.00	0.75	0.82
Habo Gard	2015	0.26	-0.94	1.45	0.66
Hoje A 14	2015	-0.05	-0.65	0.53	0.89
Hoje A 6	2015	0.20	-0.88	1.49	0.72
Hoje A 7	2015	-0.04	-1.09	0.91	0.93
Ilstorp	2015	0.00	-0.60	0.64	1.00
Lomma	2015	-0.14	-1.18	0.81	0.78
Vombs V	2015	-0.31	-0.86	0.20	0.26
Borgeby	2016	0.40	-0.15	0.98	0.17

Flackarp	2016	0.16	-0.99	1.42	0.81
Fly 30 A1	2016	-0.51	-1.50	0.54	0.33
Fly 30 A3	2016	-0.24	-1.03	0.62	0.55
Genarp	2016	-1.10	-2.04	-0.27	**0.01**
Gunnesbo	2016	0.31	-0.62	1.15	0.48
Habo Gard	2016	0.12	-1.14	1.39	0.86
Hoje A 14	2016	0.48	-0.41	1.30	0.28
Hoje A 6	2016	-0.63	-1.55	0.29	0.17
Hoje A 7	2016	0.07	-0.82	1.06	0.88
Ilstorp	2016	0.74	0.15	1.36	0.02
Lomma	2016	0.14	-1.07	1.24	0.80
Lunnarp	2016	0.01	-1.27	1.26	0.98
Vombs V	2016	0.10	-0.41	0.73	0.71

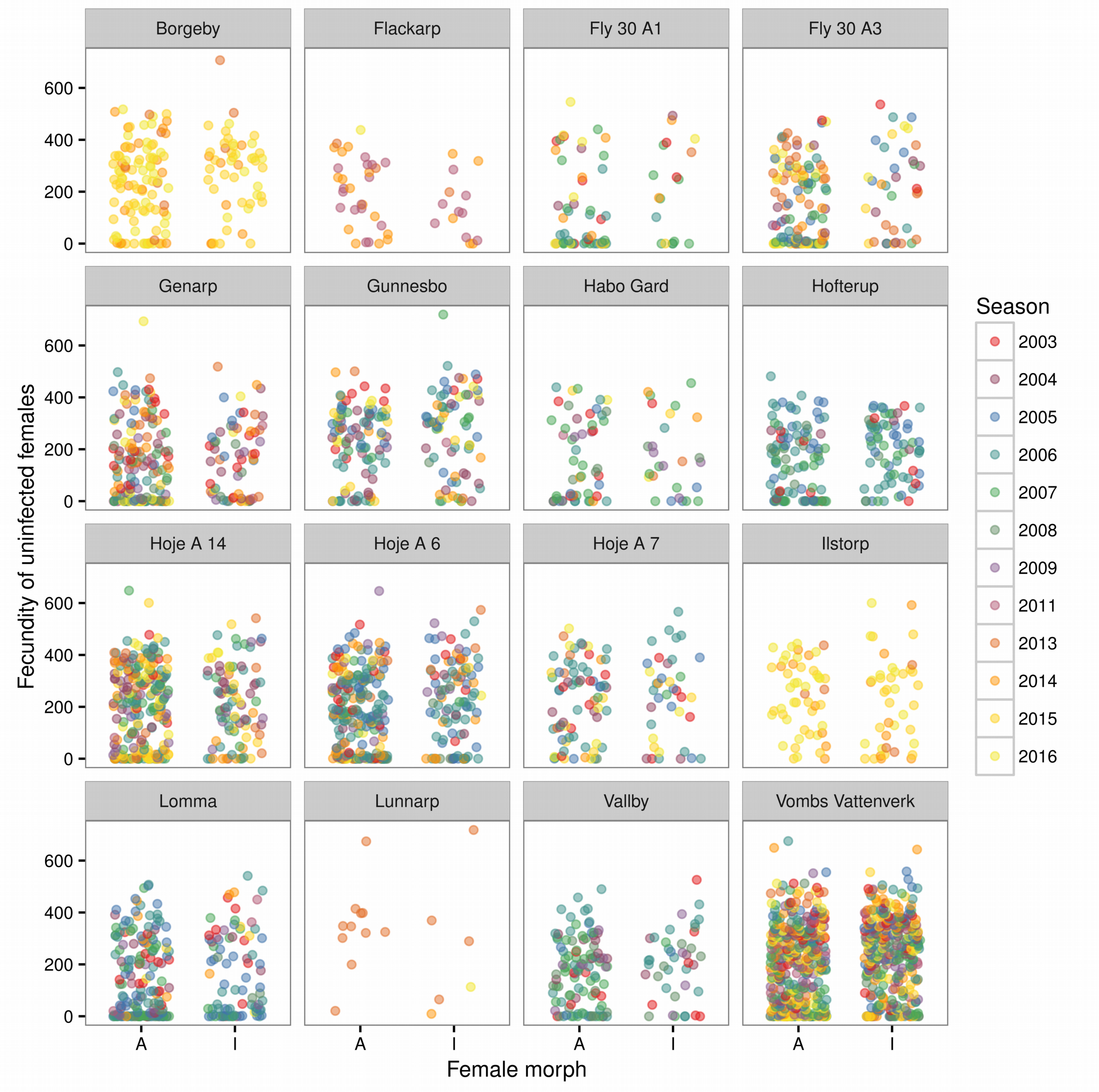

Fig S9. Fecundity of uninfected females of *I. elegans* damselflies across 16 populations and up to 14 years of field sampling. Points represent individual egg counts.
